# Preparation of quantum information encoded on three-photon decoherence-free states via cross-Kerr nonlinearities

**DOI:** 10.1038/s41598-018-32137-3

**Published:** 2018-09-14

**Authors:** Jino Heo, Min-Sung Kang, Chang Ho Hong, Jong-Phil Hong, Seong-Gon Choi

**Affiliations:** 1 College of Electrical and Computer Engineering, Chungbuk National University, Chungdae-ro 1, Seowon-Gu, Cheongju Republic of Korea; 2 Center for Quantum Information, Korea Institute of Science and Technology (KIST), Seoul, 136-791 Republic of Korea; 3 Base Technology Division, National Security Research Institute, P.O. Box 1, Yuseong, Daejeon, 34188 Republic of Korea

## Abstract

We present a scheme to encode quantum information (single logical qubit information) into three-photon decoherence-free states, which can conserve quantum information from collective decoherence, via nonlinearly optical gates (using cross-Kerr nonlinearities: XKNLs) and linearly optical devices. For the preparation of the decoherence-free state, the nonlinearly optical gates (multi-photon gates) consist of weak XKNLs, quantum bus (qubus) beams, and photon-number-resolving (PNR) measurement. Then, by using a linearly optical device, quantum information can be encoded on three-photon decoherence-free state prepared. Subsequently, by our analysis, we show that the nonlinearly optical gates using XKNLs, qubus beams, and PNR measurement are robust against the decoherence effect (photon loss and dephasing) in optical fibers. Consequently, our scheme can be experimentally implemented to efficiently generate three-photon decoherence-free state encoded quantum information, in practice.

## Introduction

In quantum information processing technologies^1–13^, quantum information carriers are the most important components, but cannot avoid being influenced by unwanted interaction (nonunitary process induced by decoherence) between the systems and the environment. Thus, for reliable quantum information processing, the researchers should try to alleviate the effect of environment-induced decoherence.

Fortunately, the methods of the active process have been researched, such as quantum error correction codes (from the concept of classical redundancy)^14–16^, dynamical decoupling controls (switching to decouple the interaction between the systems and the environment)^17–19^, and feedback controls (performing the optimal operation from the measurement result)^20–22^.

Other methods are passive processes using decoherence-free subspaces^23–37^ to overcome the effects induced by the collective decoherence^23–25^, which means that each qubit in the system is influenced by the identical decoherence. In these methods (decoherence-free subspaces), the immunity against collective decoherence is provided because the symmetrical interaction (collective decoherence) does not evolve from one subspace to another subspace in the system. Therefore, when this symmetrical interaction occurs, the quantum information encoded into the decoherence-free states can be conserved (the invariant subspace) while another subspace may be potentially changed^23–25^.

Cross-Kerr nonlinearities (XKNLs) have been utilized, both experimentally and theoretically for the implementation of quantum information processing schemes, such as the generations and preparations of entanglement^38–43^, the concentrations of entanglement^44–49^, quantum communications^5,45,50^, and the schemes of splitting quantum information^51–53^. To realize nonlinearly optical gates for generating the decoherence-free state, we use XKNLs, which have been widely researched via the indirect interaction between photons based on quantum non-demolition measurement^7,11,54–62^. Thus, by using XKNLs, several schemes^29–31,33–37^ for generating decoherence-free subspace states have been proposed to conserve quantum information encoded against collective decoherence^23–25^. However, the decoherence effect in optical fibers inevitably occurs, and the multi-qubit gates via XKNLs and homodyne measurements^33–37^ cannot avoid photon loss and dephasing under this (decoherence) effect. Therefore, the output states of nonlinearly optical gates using homodyne measurements will evolve into mixed states (decreasing fidelity) by the decoherence effect, according to previous research^55,56,62,63^. Fortunately, by apply photon-number-resolving (PNR) measurements (instead of homodyne measurement)^7,11,31,34,55,56,62,64,65^, and a displacement operator^7,55,56^ or quantum bus (qubus) beams^11,62,65^ with the increasing amplitude of the coherent state (probe beam), the decoherence effect (photon loss and dephasing) can be made arbitrarily small^55,56,62^.

In this paper, we propose a scheme that can encode quantum information into three-photon decoherence-free states (single logical qubit information) to obtain immunity against collective decoherence using nonlinearly optical gates and linearly optical devices. The designed scheme (generation of decoherence-free state) can conserve quantum information from collective decoherence (caused by evolution of symmetrical interaction^23–25^). Besides, we can prevent evolution of the output state into the mixed state induced by the decoherence effect (due to photon loss and dephasing^55,56,60,62,63^) in optical fibers by nonlinearly optical gates consisting of XKNLs, qubus beams, and PNR measurements. Finally, we show that our scheme for generating single logical qubit information (into three-photon decoherence-free states) with immunity (against collective decoherence between the system and the environment) can be experimentally implemented, and can be robust against the decoherence effect in optical fibers, through our analysis of nonlinearly optical gates using XKNLs, qubus beams, and PNR measurements.

## Single Logical Qubit Information into Three-Photon Decoherence-Free States via XKNLs and Linearly Optical Devices

For the immunity against collective decoherence, Kempe *et al*.^24^ proposed three-photon decoherence-free states carrying the logical qubits $\{|{0}_{{\rm{L}}{\rm{j}}}\rangle ,|{1}_{{\rm{Lj}}}\rangle \because {\rm{j}}={\rm{1}}\,{\rm{or}}\,{\rm{2}}\}$, as follows:

1 $$\begin{array}{ccc}|{0}_{L1}\rangle  & = & \frac{1}{\sqrt{2}}(|H\rangle |V\rangle |H\rangle -|V\rangle |H\rangle |H\rangle ),\\ |{1}_{L1}\rangle  & = & \frac{1}{\sqrt{6}}(|H\rangle |V\rangle |H\rangle +|V\rangle |H\rangle |H\rangle -2|H\rangle |H\rangle |V\rangle ),\\ |{0}_{L2}\rangle  & = & \frac{1}{\sqrt{2}}(|H\rangle |V\rangle |V\rangle -|V\rangle |H\rangle |V\rangle ),\\ |{1}_{L2}\rangle  & = & \frac{1}{\sqrt{6}}(|H\rangle |V\rangle |V\rangle +|V\rangle |H\rangle |V\rangle -2|V\rangle |V\rangle |H\rangle ),\end{array}$$

where the linearly polarized states ($|H\rangle$, horizontal; $|V\rangle$, vertical) are related to the circularly polarized states ($|R\rangle$, right; $|L\rangle$, left), such as $|H\rangle \equiv (|R\rangle +|L\rangle )/\sqrt{2}$ and $|V\rangle \equiv (|R\rangle -|L\rangle )/\sqrt{2}$. In quantum information processing schemes, we can preserve quantum information encoding into logical qubits (single logical qubit information) from the unwanted effect of collective decoherence (due to evolution of symmetrical interaction), as follows:

2 $$|{\phi }_{{\rm{Lj}}}\rangle =\alpha |{0}_{{\rm{Lj}}}\rangle +\beta |{1}_{{\rm{Lj}}}\rangle ,\,{|\alpha |}^{2}+{|\beta |}^{2}=1.$$

For reliable performance of quantum information processing, we design an optical scheme to generate three-photon (logical qubits) states, in Eq. 1, and encode single logical qubit information, in Eq. 2 (arbitrary quantum state), assisted by nonlinearly optical gates (using XKNLs, qubus beams, and PNR measurements) and linearly optical devices.

We introduce the XKNL effect (XKNL’s Hamiltonian: *H*_*Kerr*_ = *ħχN*_1_*N*_p_, where *N*_*i*_ and *χ* are the photon-number operator and the strength of nonlinearity) in Kerr medium. The interaction of Kerr (XKNL) between the photon state, ${|n\rangle }_{1}$, and the coherent state, ${|\alpha \rangle }_{{\rm{P}}}$ (photon-probe system), is given by

3 $${U}_{Kerr}{|n\rangle }_{1}{|\alpha \rangle }_{{\rm{P}}}={e}^{\frac{i}{\hslash }{H}_{Kerr}t}{|n\rangle }_{1}{|\alpha \rangle }_{{\rm{P}}}={e}^{i\theta {N}_{1}{N}_{{\rm{p}}}}{|n\rangle }_{1}{|\alpha \rangle }_{{\rm{P}}}={|n\rangle }_{1}{|\alpha {e}^{in\theta }\rangle }_{{\rm{P}}},$$

where *θ* (=*χt*) is the magnitude of the conditional phase shift for the interaction time, *t*; $|n\rangle$ is *n* photon state; and $|\alpha \rangle ={e}^{-{|\alpha |}^{2}/2}\sum _{n=0}^{\infty }\frac{{\alpha }^{n}}{\sqrt{n!}}|n\rangle$ (coherent state).

**Figure 1:**
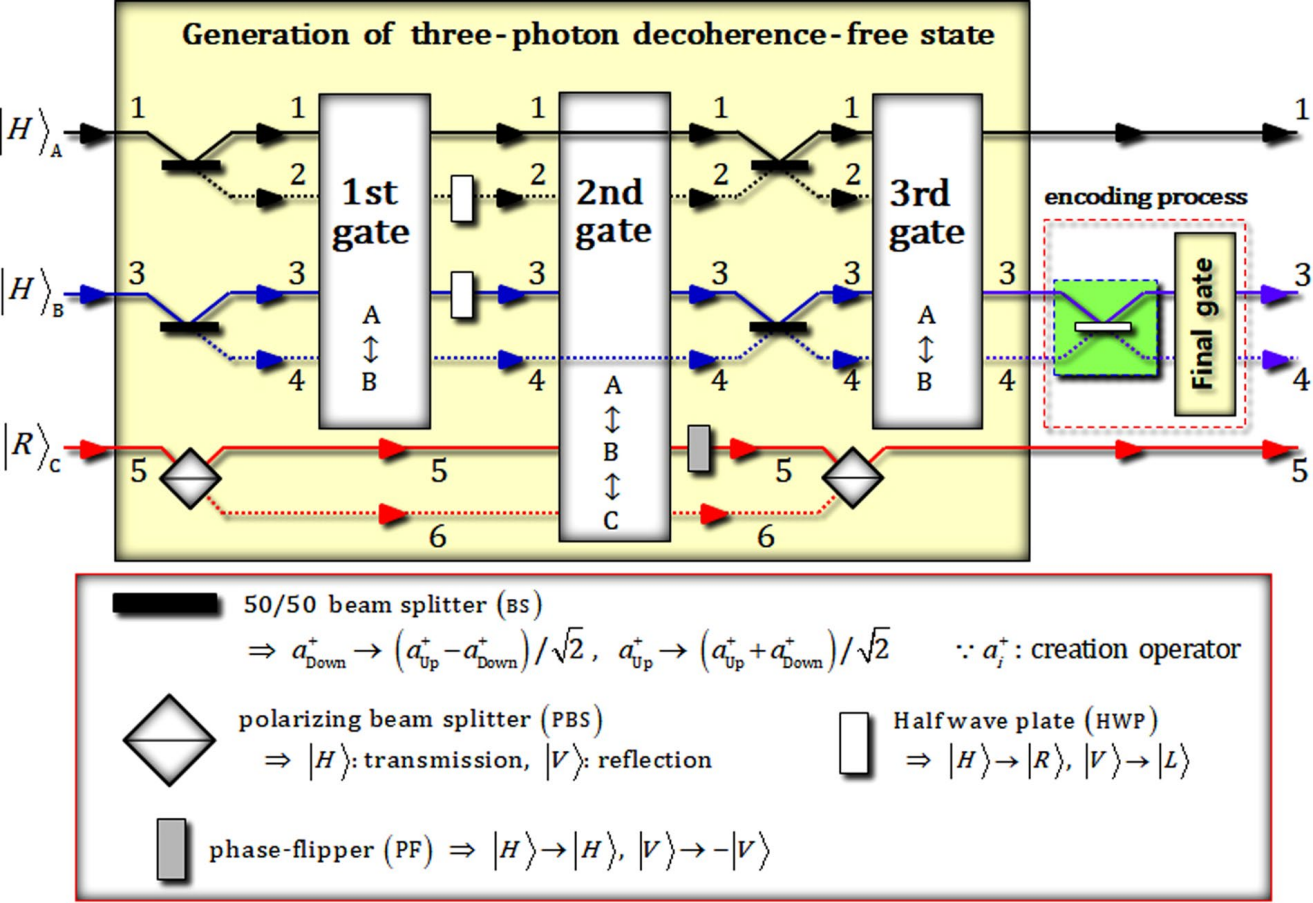
Schematic plot of single logical qubit information into three-photon decoherence-free states: This scheme consists of two parts of generation of the three-photon decoherence-free state (part 1), and encoding process (part 2). In the generation of the three-photon decoherence-free state, the three (1st, 2nd, and 3rd) gates use the nonlinearly optical effects (XKNLs). The final gate in the encoding process uses XKNLs to produce the single logical qubit information having immunity (against collective decoherence between the system and the environment).

Figure 1 shows an optical scheme to generate three-photon decoherence-free states (logical qubit as in Eq. 1) and encode quantum information (single logical qubit information as in Eq. 2) using XKNLs, qubus beams, and PNR measurements (nonlinearly optical gates) and linearly optical devices. For the protection of quantum information, this scheme can produce the single logical qubit information ($\alpha |{0}_{{\rm{L}}}\rangle +\beta |{1}_{{\rm{L}}}\rangle$) from the initial state (product state of three-photon: ${|H\rangle }_{{\rm{A}}}\otimes {|H\rangle }_{{\rm{B}}}\otimes {|R\rangle }_{{\rm{C}}}$).

First, as shown in Fig. 1, after the initial state, ${|{\psi }_{{\rm{i}}}\rangle }_{{\rm{ABC}}}={|H\rangle }_{{\rm{A}}}^{1}\otimes {|H\rangle }_{{\rm{B}}}^{3}\otimes {|R\rangle }_{{\rm{C}}}^{5}$, passes two 50/50 beam splitters (BSs) and a polarizing beam splitter (PBS), the three-photon state is transformed to

4 $${|{\psi }_{{\rm{i}}}\rangle }_{{\rm{ABC}}}\mathop{\to }\limits^{\mathrm{50}/\mathrm{50\; BSs},\mathrm{PBS}}{|{\psi }_{0}\rangle }_{{\rm{ABC}}}=\frac{1}{\sqrt{2}}({|H\rangle }_{{\rm{A}}}^{1}+{|H\rangle }_{{\rm{A}}}^{2})\otimes \frac{1}{\sqrt{2}}({|H\rangle }_{{\rm{B}}}^{3}+{|H\rangle }_{{\rm{B}}}^{4})\otimes \frac{1}{\sqrt{2}}({|V\rangle }_{{\rm{C}}}^{5}+{|H\rangle }_{{\rm{C}}}^{6}).$$

Then, the nonlinearly optical gate (1st gate) will be applied to the state, ${|{\psi }_{{\rm{0}}}\rangle }_{{\rm{ABC}}}$.

### 1^st^ gate (photons A-B)

**Figure 2:**
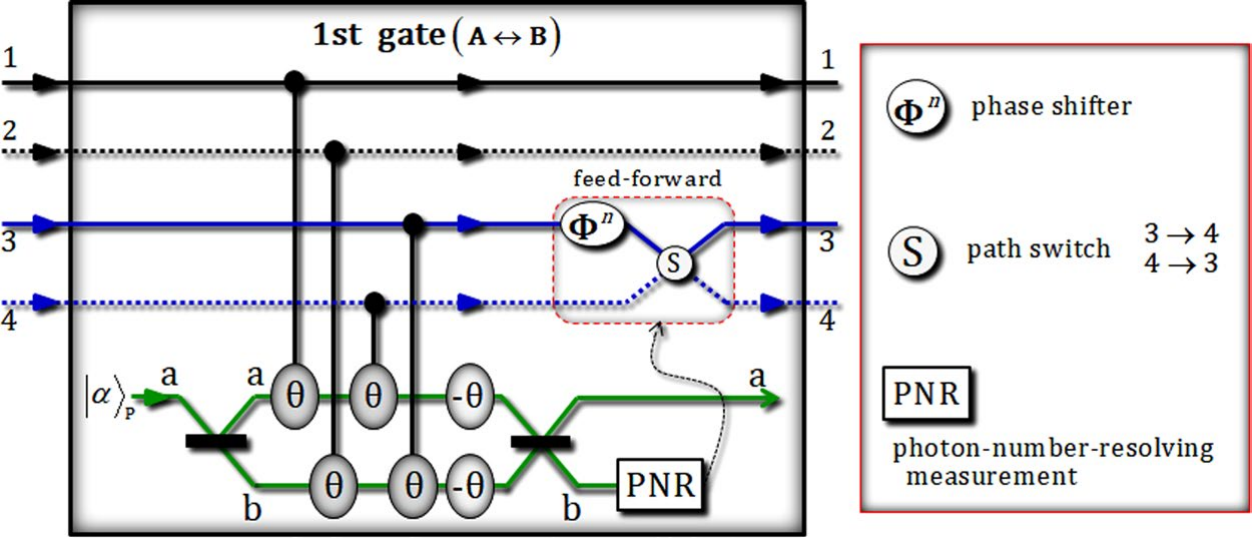
This plot represents the first gate via XKNLs, qubus beams, and PNR measurement for the interactions between two photons A and B. In the first gate, four conditional phase shifts are only positive in the qubus beams, including two linear phase shifts. After PNR measurement, according to the result of the measurement, feed-forward (phase shift **Φ**^*n*^ and path switch S) is or is not operated on photon B.

The first gate (in Fig. 2) consists of four conditional phase shifts θ (by XKNLs: positive), two linear phase shifts −θ (negative), qubus beams (two 50/50 BSs and PNR measurement), and feed-forward (phase shifter **Φ**^*n*^ and path switch S).

After the operation of the first gate to the state ${|{\psi }_{{\rm{0}}}\rangle }_{{\rm{ABC}}}$, the output state ${|{\psi ^{\prime} }_{0}\rangle }_{{\rm{ABC}}}$ of premeasurement (PNR) is given by

5 $$\begin{array}{rcl}{||{\psi ^{\prime} }_{0}\rangle \rangle }_{{\rm{ABC}}} & = & [{|\alpha \rangle }_{{\rm{P}}}^{{\rm{a}}}\otimes \frac{1}{\sqrt{2}}(\frac{1}{\sqrt{2}}{|H\rangle }_{{\rm{A}}}^{1}{|H\rangle }_{{\rm{B}}}^{3}+\frac{1}{\sqrt{2}}{|H\rangle }_{{\rm{A}}}^{2}{|H\rangle }_{{\rm{B}}}^{4})\otimes {|0\rangle }_{{\rm{P}}}^{{\rm{b}}}\\  &  & +\,{|\alpha \,\cos \,{\rm{\theta }}\rangle }_{{\rm{P}}}^{{\rm{a}}}\otimes \frac{1}{\sqrt{2}}{e}^{-\frac{{(\alpha \sin {\rm{\theta }})}^{2}}{2}}\sum _{n=0}^{\infty }\frac{{(i\alpha \sin {\rm{\theta }})}^{n}}{\sqrt{n!}}\\  &  & \times \,(\frac{1}{\sqrt{2}}{|H\rangle }_{{\rm{A}}}^{1}{|H\rangle }_{{\rm{B}}}^{4}+\frac{{(-1)}^{n}}{\sqrt{2}}{|H\rangle }_{{\rm{A}}}^{2}{|H\rangle }_{{\rm{B}}}^{3})\otimes {|n\rangle }_{{\rm{P}}}^{{\rm{b}}}]\otimes \frac{1}{\sqrt{2}}({|V\rangle }_{{\rm{C}}}^{5}+{|H\rangle }_{{\rm{C}}}^{6}),\end{array}$$

where $${|\alpha \rangle }_{{\rm{P}}}$$ is the probe beam (coherent state). The interaction of 50/50 BS in qubus beams is expressed as $${|\alpha \rangle }^{{\rm{a}}}{|\beta \rangle }^{{\rm{b}}}\mathop{\to }\limits^{{\rm{BS}}}{|(\alpha +\beta )/\sqrt{2}\rangle }^{{\rm{a}}}{|(\alpha -\beta )/\sqrt{2}\rangle }^{{\rm{b}}}$$, and $$|\pm i\alpha \,\sin \,\theta \rangle ={e}^{-\frac{{(\alpha \sin \theta )}^{2}}{2}}\sum _{n=0}^{\infty }\frac{{(\pm i\alpha \sin \theta )}^{n}}{\sqrt{n!}}|n\rangle $$ for *α* ∈ **R**. When we measure the qubus beam of path b using PNR measurement, if the result is $${|0\rangle }_{{\rm{P}}}^{{\rm{b}}}$$ (no detection), we can acquire the state as $$({|H\rangle }_{{\rm{A}}}^{1}{|H\rangle }_{{\rm{B}}}^{3}+{|H\rangle }_{{\rm{A}}}^{2}{|H\rangle }_{{\rm{B}}}^{4})/\sqrt{2}\otimes $$$$({|V\rangle }_{{\rm{C}}}^{5}+{|H\rangle }_{{\rm{C}}}^{6})/\sqrt{2}$$. On the other hand, if the result is $${|n\rangle }_{{\rm{P}}}^{{\rm{b}}}$$ (*n* ≠ 0), we can transform the resulting state of $${|n\rangle }_{{\rm{P}}}^{{\rm{b}}}$$ to the resulting state of $${|0\rangle }_{{\rm{P}}}^{{\rm{b}}}$$, $$({|H\rangle }_{{\rm{A}}}^{1}{|H\rangle }_{{\rm{B}}}^{3}+{|H\rangle }_{{\rm{A}}}^{2}{|H\rangle }_{{\rm{B}}}^{4})/\sqrt{2}\otimes $$$$({|V\rangle }_{{\rm{C}}}^{5}+{|H\rangle }_{{\rm{C}}}^{6})/\sqrt{2}$$, by feed-forward (phase shifter **Φ**^*n*^ and path switch S), according to the result, *n*, as described in Fig. 2. The error probability $${{\rm{P}}}_{{\rm{err}}}^{{\rm{1st}}}$$ of the first gate can be calculated as $${{\rm{P}}}_{{\rm{err}}}^{{\rm{1st}}}=({e}^{-{\alpha }^{2}{\sin }^{2}{\rm{\theta }}})/2\approx ({e}^{-{\alpha }^{2}{{\rm{\theta }}}^{2}})/2$$, due to the probability to measure $${|0\rangle }_{{\rm{P}}}^{{\rm{b}}}$$ (no detection) in $${|\pm i\alpha \,\sin \,\theta \rangle }_{{\rm{P}}}^{{\rm{b}}}$$ on path b, where $${\sin }^{2}{\rm{\theta }}\approx {{\rm{\theta }}}^{2}$$ for $$\alpha \gg 1$$ and $${\rm{\theta }}\ll 1$$. Thus, we can obtain $${{\rm{P}}}_{{\rm{err}}}^{{\rm{1st}}} < {10}^{-3}$$ for the reliable performance of the first gate for the fixed parameters (the amplitude of the coherent state and the magnitude of condition phase shift) as *α*θ = 2.5. Subsequently, two half wave plates (HWPs), in Fig. 1, on path 2 and 3 are operated to the output state of the first gate, as follows:

6 $$\begin{array}{c}{|{\psi }_{0}\rangle }_{{\rm{ABC}}}\mathop{\to }\limits^{{\rm{1st}}\,{\rm{gate}}}\frac{1}{\sqrt{2}}({|H\rangle }_{{\rm{A}}}^{1}{|H\rangle }_{{\rm{B}}}^{3}+{|H\rangle }_{{\rm{A}}}^{2}{|H\rangle }_{{\rm{B}}}^{4})\otimes \frac{1}{\sqrt{2}}({|V\rangle }_{{\rm{C}}}^{5}+{|H\rangle }_{{\rm{C}}}^{6})\,\mathop{\to }\limits^{{\rm{HWPs}}}\\ \to {|{\psi }_{1}\rangle }_{{\rm{ABC}}}=\frac{1}{2}({|H\rangle }_{{\rm{A}}}^{1}{|H\rangle }_{{\rm{B}}}^{3}+{|H\rangle }_{{\rm{A}}}^{1}{|V\rangle }_{{\rm{B}}}^{3}+{|H\rangle }_{{\rm{A}}}^{2}{|H\rangle }_{{\rm{B}}}^{4}+{|V\rangle }_{{\rm{A}}}^{2}{|H\rangle }_{{\rm{B}}}^{4})\otimes \frac{1}{\sqrt{2}}({|V\rangle }_{{\rm{C}}}^{5}+{|H\rangle }_{{\rm{C}}}^{6}).\end{array}$$

### 2nd gate (photons A-B-C)

**Figure 3:**
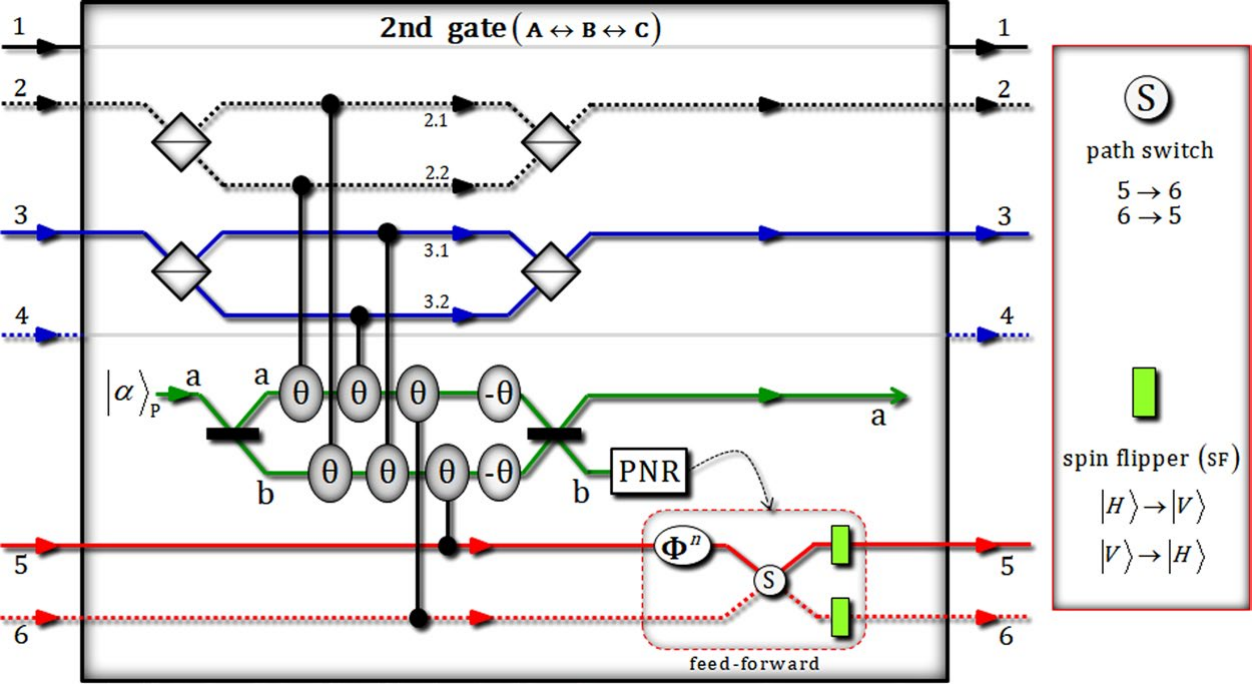
This plot represents the second gate via XKNLs, qubus beams, and PNR measurement for the interactions between photons A, B, and C. In the second gate, six conditional phase shifts are only positive in the qubus beams, including two linear phase shifts. After PNR measurement, according to the result of the measurement, feed-forward (phase shift **Φ**^*n*^, path switch S, and two spin flippers) is or is not operated on photon C.

The second gate consists of four PBSs, six conditional phase shifts θ (by XKNLs: positive), two linear phase shifts −θ (negative), qubus beams (two 50/50 BSs and PNR measurement), and feed-forward (phase shifter **Φ**^*n*^, path switch S, and two spin flippers), as shown in Fig. 3.

After the operation of the second gate to the state ${|{\psi }_{{\rm{1}}}\rangle }_{{\rm{ABC}}}$ in Eq. 6, the output state ${|{\psi }_{1}^{\prime} \rangle }_{{\rm{ABC}}}$ of premeasurement (PNR) is given by

7 $$\begin{array}{ccc}{|{\psi {\rm{^{\prime} }}}_{1}\rangle }_{{\rm{A}}{\rm{B}}{\rm{C}}} & = & {|\alpha \rangle }_{{\rm{P}}}^{{\rm{a}}}\otimes \frac{1}{\sqrt{2}}[\frac{1}{2}({|H\rangle }_{{\rm{A}}}^{1}{|H\rangle }_{{\rm{B}}}^{3}{|V\rangle }_{{\rm{C}}}^{5}+{|H\rangle }_{{\rm{A}}}^{1}{|V\rangle }_{{\rm{B}}}^{3}{|H\rangle }_{{\rm{C}}}^{6}\\  &  & +\,{|H\rangle }_{{\rm{A}}}^{2}{|H\rangle }_{{\rm{B}}}^{4}{|V\rangle }_{{\rm{C}}}^{5}+{|V\rangle }_{{\rm{A}}}^{2}{|H\rangle }_{{\rm{B}}}^{4}{|H\rangle }_{{\rm{C}}}^{6})]\,\\  &  & +\,\otimes {|0\rangle }_{{\rm{P}}}^{{\rm{b}}}+{|\alpha \,\cos \,\theta \rangle }_{{\rm{P}}}^{{\rm{a}}}\otimes \frac{1}{\sqrt{2}}{e}^{-\frac{{(\alpha \sin \theta )}^{2}}{2}}\sum _{n=0}^{{\rm{\infty }}}\frac{{(i\alpha \sin \theta )}^{n}}{\sqrt{n!}}\\  &  & \times \,[\frac{1}{2}({|H\rangle }_{{\rm{A}}}^{1}{|H\rangle }_{{\rm{B}}}^{3}{|H\rangle }_{{\rm{C}}}^{6}+{|H\rangle }_{{\rm{A}}}^{2}{|H\rangle }_{{\rm{B}}}^{4}{|H\rangle }_{{\rm{C}}}^{6}+{(-1)}^{n}{|H\rangle }_{{\rm{A}}}^{1}{|V\rangle }_{{\rm{B}}}^{3}{|V\rangle }_{{\rm{C}}}^{5}\\  &  & +\,{(-1)}^{n}{|V\rangle }_{{\rm{A}}}^{2}{|H\rangle }_{{\rm{B}}}^{4}{|V\rangle }_{{\rm{C}}}^{5})]\otimes {|n\rangle }_{{\rm{P}}}^{{\rm{b}}}.\end{array}$$

Then, if the result of measurement (in the qubus beam of path b) is ${|0\rangle }_{{\rm{P}}}^{{\rm{b}}}$ (no detection), the state, ${|{\psi ^{\prime} }_{1}\rangle }_{{\rm{ABC}}}$, of Eq. 7 is collapsed to $\frac{1}{2}({|H\rangle }_{{\rm{A}}}^{1}{|H\rangle }_{{\rm{B}}}^{3}{|V\rangle }_{{\rm{C}}}^{5}+{|H\rangle }_{{\rm{A}}}^{1}{|V\rangle }_{{\rm{B}}}^{3}{|H\rangle }_{{\rm{C}}}^{6}+{|H\rangle }_{{\rm{A}}}^{2}{|H\rangle }_{{\rm{B}}}^{4}{|V\rangle }_{{\rm{C}}}^{5}+{|V\rangle }_{{\rm{A}}}^{2}{|H\rangle }_{{\rm{B}}}^{4}{|H\rangle }_{{\rm{C}}}^{6})$. On the other hand, if the result is ${|n\rangle }_{{\rm{P}}}^{{\rm{b}}}$ (*n* ≠ 0), we can transform the resulting state of ${|n\rangle }_{{\rm{P}}}^{{\rm{b}}}$ to the resulting state of ${|0\rangle }_{{\rm{P}}}^{{\rm{b}}}$ by feed-forward (phase shifter **Φ**^*n*^, path switch S, and two spin flippers), according to the result, as described in Fig. 3. Also, the error probability ${{\rm{P}}}_{{\rm{err}}}^{{\rm{2nd}}}$ of the second gate is identical with the error probability ${{\rm{P}}}_{{\rm{err}}}^{{\rm{1st}}}$ of the first gate as ${{\rm{P}}}_{{\rm{err}}}^{{\rm{2nd}}}=$ ${{\rm{P}}}_{{\rm{err}}}^{{\rm{1st}}}\approx ({e}^{-{\alpha }^{2}{{\rm{\theta }}}^{2}})/2$ ($\alpha {\rm{\theta }}=2.5\Rightarrow {{\rm{P}}}_{{\rm{err}}}^{{\rm{2nd}}} < {10}^{-3}$). Subsequently, two 50/50 BSs (photon A and B), phase-flipper (PF), and PBS (photon C) in Fig. 1 are applied to the output state of the second gate, as follows:

8 $$\begin{array}{ccc}{|{\psi }_{1}\rangle }_{{\rm{A}}{\rm{B}}{\rm{C}}} & \mathop{\to }\limits^{2{\rm{n}}{\rm{d}}\,{\rm{g}}{\rm{a}}{\rm{t}}{\rm{e}}} & \frac{1}{2}({|H\rangle }_{{\rm{A}}}^{1}{|H\rangle }_{{\rm{B}}}^{3}{|V\rangle }_{{\rm{C}}}^{5}+{|H\rangle }_{{\rm{A}}}^{1}{|V\rangle }_{{\rm{B}}}^{3}{|H\rangle }_{{\rm{C}}}^{6}\\  &  & +\,{|H\rangle }_{{\rm{A}}}^{2}{|H\rangle }_{{\rm{B}}}^{4}{|V\rangle }_{{\rm{C}}}^{5}+{|V\rangle }_{{\rm{A}}}^{2}{|H\rangle }_{{\rm{B}}}^{4}{|H\rangle }_{{\rm{C}}}^{6})\,\mathop{\to }\limits^{50/50\,{\rm{B}}{\rm{S}}{\rm{s}},\,{\rm{P}}{\rm{F}},\,{\rm{P}}{\rm{B}}{\rm{S}}}\\  & \to {|{\psi }_{2}\rangle }_{{\rm{A}}{\rm{B}}{\rm{C}}}\,= & \frac{1}{\sqrt{2}}[\frac{1}{2\sqrt{2}}({|H\rangle }_{{\rm{A}}}^{2}{|V\rangle }_{{\rm{B}}}^{3}{|H\rangle }_{{\rm{C}}}^{5}-{|V\rangle }_{{\rm{A}}}^{2}{|H\rangle }_{{\rm{B}}}^{3}{|H\rangle }_{{\rm{C}}}^{5})\\  &  & +\,\frac{1}{2\sqrt{2}}({|H\rangle }_{{\rm{A}}}^{1}{|V\rangle }_{{\rm{B}}}^{3}{|H\rangle }_{{\rm{C}}}^{5}+{|V\rangle }_{{\rm{A}}}^{1}{|H\rangle }_{{\rm{B}}}^{3}{|H\rangle }_{{\rm{C}}}^{5}-2{|H\rangle }_{{\rm{A}}}^{1}{|H\rangle }_{{\rm{B}}}^{3}{|V\rangle }_{{\rm{C}}}^{5})\\  &  & +\,\frac{1}{2\sqrt{2}}({|H\rangle }_{{\rm{A}}}^{1}{|V\rangle }_{{\rm{B}}}^{4}{|H\rangle }_{{\rm{C}}}^{5}-{|V\rangle }_{{\rm{A}}}^{1}{|H\rangle }_{{\rm{B}}}^{4}{|H\rangle }_{{\rm{C}}}^{5})\\  &  & +\,\frac{1}{2\sqrt{2}}({|H\rangle }_{{\rm{A}}}^{2}{|V\rangle }_{{\rm{B}}}^{4}{|H\rangle }_{{\rm{C}}}^{5}+{|V\rangle }_{{\rm{A}}}^{2}{|H\rangle }_{{\rm{B}}}^{4}{|H\rangle }_{{\rm{C}}}^{5}-2{|H\rangle }_{{\rm{A}}}^{2}{|H\rangle }_{{\rm{B}}}^{4}{|V\rangle }_{{\rm{C}}}^{5})]\\  &  & \equiv \frac{1}{\sqrt{2}}[(\frac{1}{2}{|{0}_{{\rm{L}}1}\rangle }^{235}+\frac{\sqrt{3}}{2}{|{1}_{{\rm{L}}1}\rangle }^{135})+(\frac{1}{2}{|{0}_{{\rm{L}}1}\rangle }^{145}+\frac{\sqrt{3}}{2}{|{1}_{{\rm{L}}1}\rangle }^{245})].\end{array}$$

Also, if we want the state ${|{\psi }_{2}\rangle }_{{\rm{ABC}}}$, in Eq. 8, to be the superposition state on the basis of $\{|{0}_{{\rm{L}}{\rm{2}}}\rangle ,|{1}_{{\rm{L}}{\rm{2}}}\rangle \}$, as in Eq. 1, we can transform to apply the spin flippers on all paths (1, 2, 3, 4, and 5).

### 3rd gate (photons A-B)

**Figure 4:**
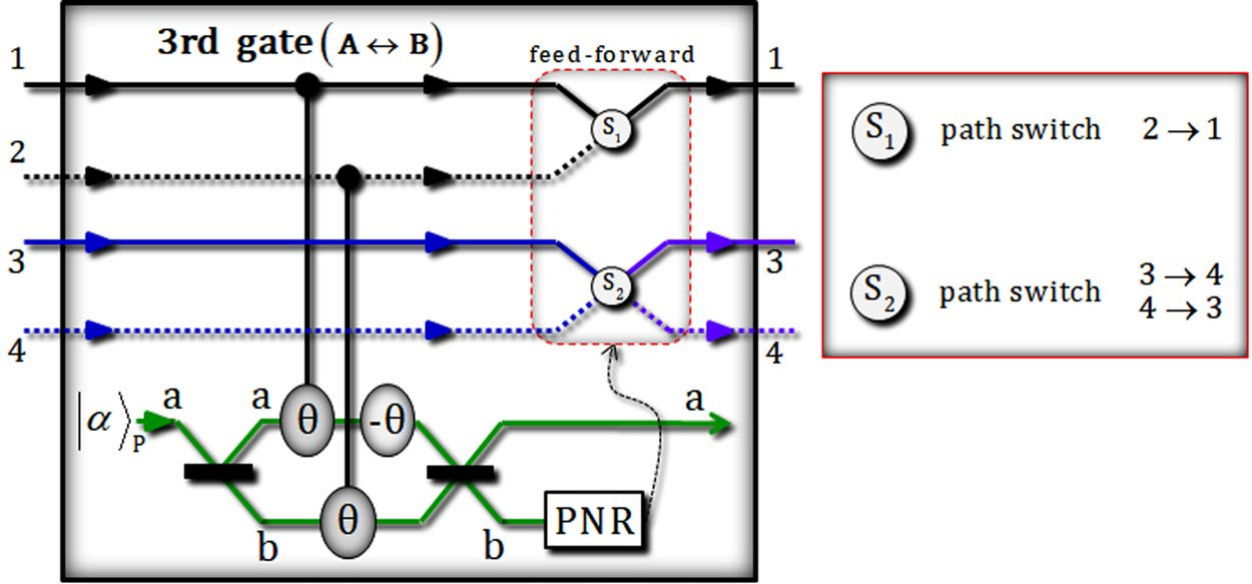
This plot represents the third gate via XKNLs, qubus beams, and PNR measurement for the interactions between two photons A and B. In the third gate, two conditional phase shifts are only positive, in the qubus beams, including one linear phase shift. After PNR measurement, according to the result of the measurement, feed-forward (two path switches S_1_ and S_2_) is or is not operated on photons A and B.

For the merging of paths (1 and 2), the third gate consists of two conditional phase shifts θ (by XKNLs: positive), a linear phase shift −θ (negative), qubus beams (two 50/50 BSs and PNR measurement), and feed-forward (two path switches S_1_ and S_2_), as described in Fig. 4. After the operation of the third gate to the state ${|{\psi }_{{\rm{2}}}\rangle }_{{\rm{ABC}}}$ in Eq. 8, the output state ${|{\psi ^{\prime} }_{2}\rangle }_{{\rm{ABC}}}$ of premeasurement (PNR) is given by

9 $$\begin{array}{rcl}{|{\psi ^{\prime} }_{{\rm{2}}}\rangle }_{{\rm{ABC}}} & = & {|\alpha \rangle }_{{\rm{P}}}^{{\rm{a}}}\otimes \frac{1}{\sqrt{2}}(\frac{1}{2}{|{0}_{{\rm{L1}}}\rangle }^{145}+\frac{\sqrt{3}}{2}{|{1}_{{\rm{L1}}}\rangle }^{135})\otimes {|0\rangle }_{{\rm{P}}}^{{\rm{b}}}+{|\alpha \,\cos \,{\rm{\theta }}\rangle }_{{\rm{P}}}^{{\rm{a}}}\\  &  & \otimes \,\frac{1}{\sqrt{2}}{e}^{-\frac{{(\alpha \sin {\rm{\theta }})}^{2}}{2}}\sum _{n=0}^{\infty }\frac{{(-i\alpha \sin {\rm{\theta }})}^{n}}{\sqrt{n!}}(\frac{1}{2}{|{0}_{{\rm{L1}}}\rangle }^{235}+\frac{\sqrt{3}}{2}{|{1}_{{\rm{L1}}}\rangle }^{245})\otimes {|n\rangle }_{{\rm{P}}}^{{\rm{b}}}.\end{array}$$

When we measure the qubus beam of path b using PNR measurement, if the result is ${|0\rangle }_{{\rm{P}}}^{{\rm{b}}}$ (no detection), we can show the state as $({|{0}_{{\rm{L1}}}\rangle }^{145}+\sqrt{3}{|{1}_{{\rm{L1}}}\rangle }^{135})/2$. On the other hand, if the result is ${|n\rangle }_{{\rm{P}}}^{{\rm{b}}}$ (*n* ≠ 0), we can switch the path of state in the result ${|n\rangle }_{{\rm{P}}}^{{\rm{b}}}$, $({|{0}_{{\rm{L}}{\rm{1}}}\rangle }^{235}+\sqrt{3}{|{1}_{{\rm{L1}}}\rangle }^{245})/2$, to the state of result ${|0\rangle }_{{\rm{P}}}^{{\rm{b}}}$, $({|{0}_{{\rm{L}}{\rm{1}}}\rangle }^{145}+\sqrt{3}{|{1}_{{\rm{L}}{\rm{1}}}\rangle }^{135})/2$, by feed-forward (path switches S_1_ and S_2_), according to the result *n* ≠ 0, as described in Fig. 4. The error probability ${{\rm{P}}}_{{\rm{err}}}^{{\rm{3rd}}}$ of the third gate is also the same as the error probabilities of the first and second gates, as ${{\rm{P}}}_{{\rm{err}}}^{{\rm{1st}}}={{\rm{P}}}_{{\rm{err}}}^{{\rm{2nd}}}=$ ${{\rm{P}}}_{{\rm{err}}}^{{\rm{3rd}}}\approx ({e}^{-{\alpha }^{2}{{\rm{\theta }}}^{2}})/2$.

Consequently, the output state ${|{\psi }_{{\rm{3}}}\rangle }_{{\rm{ABC}}}$ (the superposition of logical qubits by three-photon decoherence-free states) from the scheme of generation of three-photon decoherence-free states is expressed as

10 $${|{\psi }_{3}\rangle }_{{\rm{ABC}}}=\frac{1}{2}{|{0}_{{\rm{L1}}}\rangle }^{145}+\frac{\sqrt{3}}{2}{|{1}_{{\rm{L1}}}\rangle }^{135},$$

where it is also possible for the output state ${|{\psi }_{{\rm{3}}}\rangle }_{{\rm{ABC}}}$ to transform to the superposition state on the basis of $\{|{0}_{{\rm{L2}}}\rangle ,|{1}_{{\rm{L}}{\rm{2}}}\rangle \}$ by the operation of spin flippers on paths.

### Encoding process (photon B)

**Figure 5:**
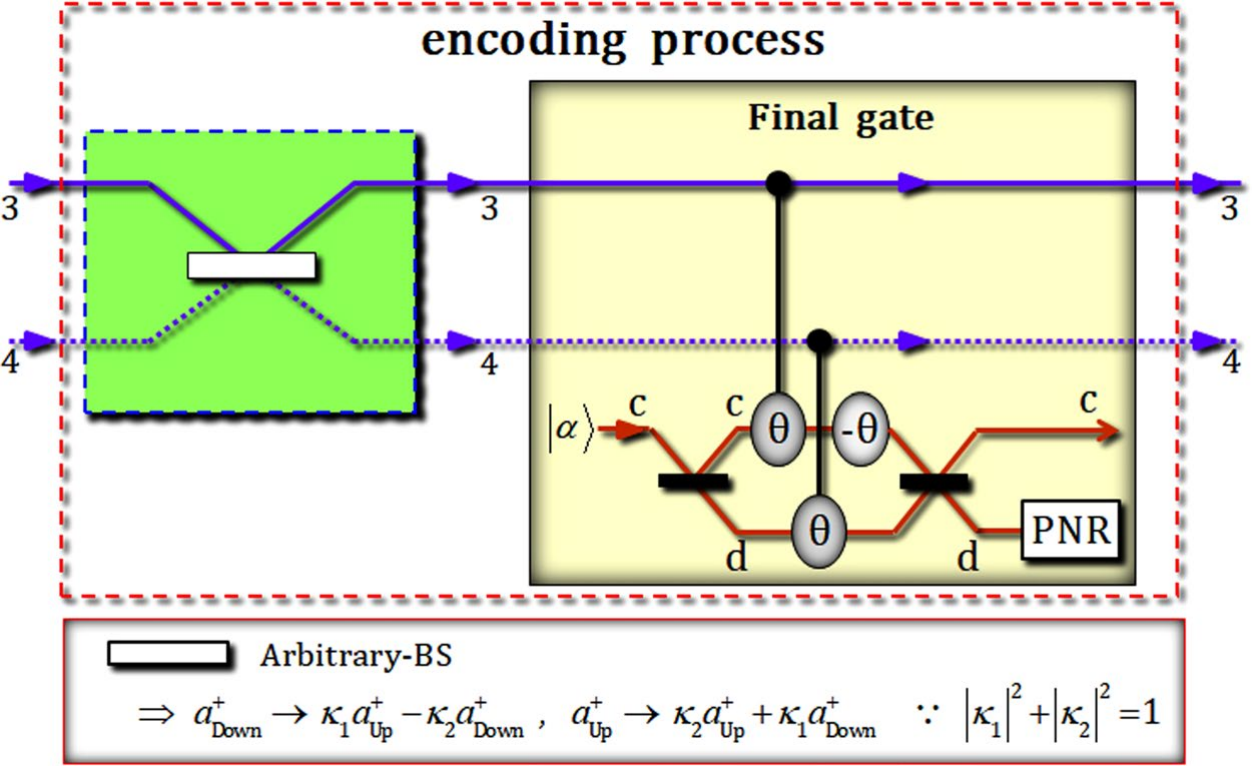
This plot represents the encoding process using a linearly optical device (arbitrary-BS) and nonlinearly optical gate (final gate: via XKNLs, qubus beams, and PNR measurement). For encoding the arbitrary quantum state into the three-photon decoherence-free states (single logical qubit information), arbitrary-BS, which can perform the reflection and transmission with the arbitrary probabilities, is applied to photon 2 in the linearly optical part. Then the final gate is used to arrange the paths of photon 2 into the merged single path.

The encoding process consists of an arbitrary-BS (linear part), two conditional phase shifts θ (by XKNLs: positive), one linear phase shift −θ (negative), in qubus beams (nonlinear part), as shown in Fig. 5.

After the state ${|{\psi }_{{\rm{E}}}\rangle }_{{\rm{ABC}}}$, in Eq. 10, passes through the arbitrary-BS for encoding the arbitrary quantum information [random or prearranged values (*α*_*i*_ and *β*_*i*_) by arbitrary-BS] into the three-photon decoherence-free states (single logical qubit information), the state ${|{\psi }_{{\rm{E}}}\rangle }_{{\rm{ABC}}}$ encoded the arbitrary information is given by

11 $$\begin{array}{c}\begin{array}{ccc}{|{\psi }_{3}\rangle }_{{\rm{A}}{\rm{B}}{\rm{C}}} & \mathop{\to }\limits^{{\rm{A}}{\rm{r}}{\rm{b}}{\rm{i}}{\rm{t}}{\rm{r}}{\rm{a}}{\rm{r}}{\rm{y}}-{\rm{B}}{\rm{S}}} & \end{array}\\ \begin{array}{ccc}\to {|{\psi }_{{\rm{E}}}\rangle }_{{\rm{A}}{\rm{B}}{\rm{C}}} & = & \frac{1}{\sqrt{2}}[\frac{1}{\sqrt{{|{\kappa }_{1}|}^{2}+3{|{\kappa }_{2}|}^{2}}}({\kappa }_{1}{|{0}_{{\rm{L}}1}\rangle }^{135}+\sqrt{3}{\kappa }_{2}{|{1}_{{\rm{L}}1}\rangle }^{135})\\  &  & +\,\frac{1}{\sqrt{{|{\kappa }_{2}|}^{2}+3{|{\kappa }_{1}|}^{2}}}(\,-\,{\kappa }_{2}{|{0}_{{\rm{L}}1}\rangle }^{145}+\sqrt{3}{\kappa }_{1}{|{1}_{{\rm{L}}1}\rangle }^{145})]\\  &  & \equiv \frac{1}{\sqrt{2}}[({\alpha }_{1}{|{0}_{{\rm{L}}1}\rangle }^{135}+{\beta }_{2}{|{1}_{{\rm{L}}1}\rangle }^{135})+({\alpha }_{2}{|{0}_{{\rm{L}}1}\rangle }^{145}+{\beta }_{2}{|{1}_{{\rm{L}}1}\rangle }^{145})].\end{array}\end{array}$$

where the transmission rate (*κ*_1_) and reflection rate (*κ*_2_) of the arbitrary-BS can be adjusted for our purposes (communication, information transfer, computation, etc.), and |*α*_1_|^2^ + |*β*_1_|^2^ = |*α*_2_|^2^ + |*β*_2_|^2^ = 1. Then, after the operation of the final gate to the state ${|{\psi }_{{\rm{E}}}\rangle }_{{\rm{ABC}}}$ in Eq. 11, the output state ${|{\psi ^{\prime} }_{{\rm{E}}}\rangle }_{{\rm{ABC}}}$ of premeasurement (PNR) is given by

12 $$\begin{array}{rcl}{|{\psi ^{\prime} }_{{\rm{E}}}\rangle }_{{\rm{ABC}}} & = & {|\alpha \rangle }^{{\rm{a}}}\otimes \frac{1}{\sqrt{2}}({\alpha }_{1}{|{0}_{{\rm{L1}}}\rangle }^{135}+{\beta }_{1}{|{1}_{{\rm{L1}}}\rangle }^{135})\otimes {|0\rangle }^{{\rm{b}}}+{|\alpha \cos {\rm{\theta }}\rangle }^{{\rm{a}}}\\  &  & \otimes \frac{1}{\sqrt{2}}{e}^{-\frac{{(\alpha \sin {\rm{\theta }})}^{2}}{2}}\sum _{n=0}^{\infty }\frac{{(-i\alpha \sin {\rm{\theta }})}^{n}}{\sqrt{n!}}({\alpha }_{2}{|{0}_{{\rm{L1}}}\rangle }^{145}+{\beta }_{2}{|{1}_{{\rm{L1}}}\rangle }^{145})\otimes {|n\rangle }^{{\rm{b}}}.\end{array}$$

Here, also, the error probability ${{\rm{P}}}_{{\rm{err}}}^{{\rm{Fin}}}$ of the final gate will be calculated as ${{\rm{P}}}_{{\rm{err}}}^{{\rm{1st}}}={{\rm{P}}}_{{\rm{err}}}^{{\rm{2nd}}}={{\rm{P}}}_{{\rm{err}}}^{{\rm{3rd}}}\approx ({e}^{-{\alpha }^{2}{{\rm{\theta }}}^{2}})/2$ after PNR measurement on path b. Subsequently, if the probe beam on path b is measured by PNR measurement, the final state (single logical qubit information on three-photon decoherence-free states in Eq. 2) is generated in accordance with the measurement outcome (PNR) with the error probability ${{\rm{P}}}_{{\rm{err}}}^{{\rm{Fin}}}$($={{\rm{P}}}_{{\rm{err}}}^{{\rm{1st}}}={{\rm{P}}}_{{\rm{err}}}^{{\rm{2nd}}}={{\rm{P}}}_{{\rm{err}}}^{{\rm{3rd}}}$), as follows:

13 $$\begin{array}{c}(n=0)\Rightarrow {|{\phi }_{{\rm{L1}}}\rangle }_{{\rm{ABC}}}={\alpha }_{1}{|{0}_{{\rm{L1}}}\rangle }^{135}+{\beta }_{1}{|{1}_{{\rm{L1}}}\rangle }^{135},\\ (n\ne 0)\Rightarrow {|{\phi ^{\prime} }_{{\rm{L1}}}\rangle }_{{\rm{ABC}}}={\alpha }_{2}{|{0}_{{\rm{L1}}}\rangle }^{145}+{\beta }_{2}{|{1}_{{\rm{L1}}}\rangle }^{145},\end{array}$$

where the final state ${|{\phi ^{\prime} }_{{\rm{L}}1}\rangle }_{{\rm{ABC}}}$ can be transformed to the state ${|{\phi }_{{\rm{L}}{\rm{1}}}\rangle }_{{\rm{ABC}}}$ by performing the rotation or unitary operations because of the setting parameters (*κ*_1_ and *κ*_2_) in the arbitrary-BS, and vice versa.

**Figure 6:**
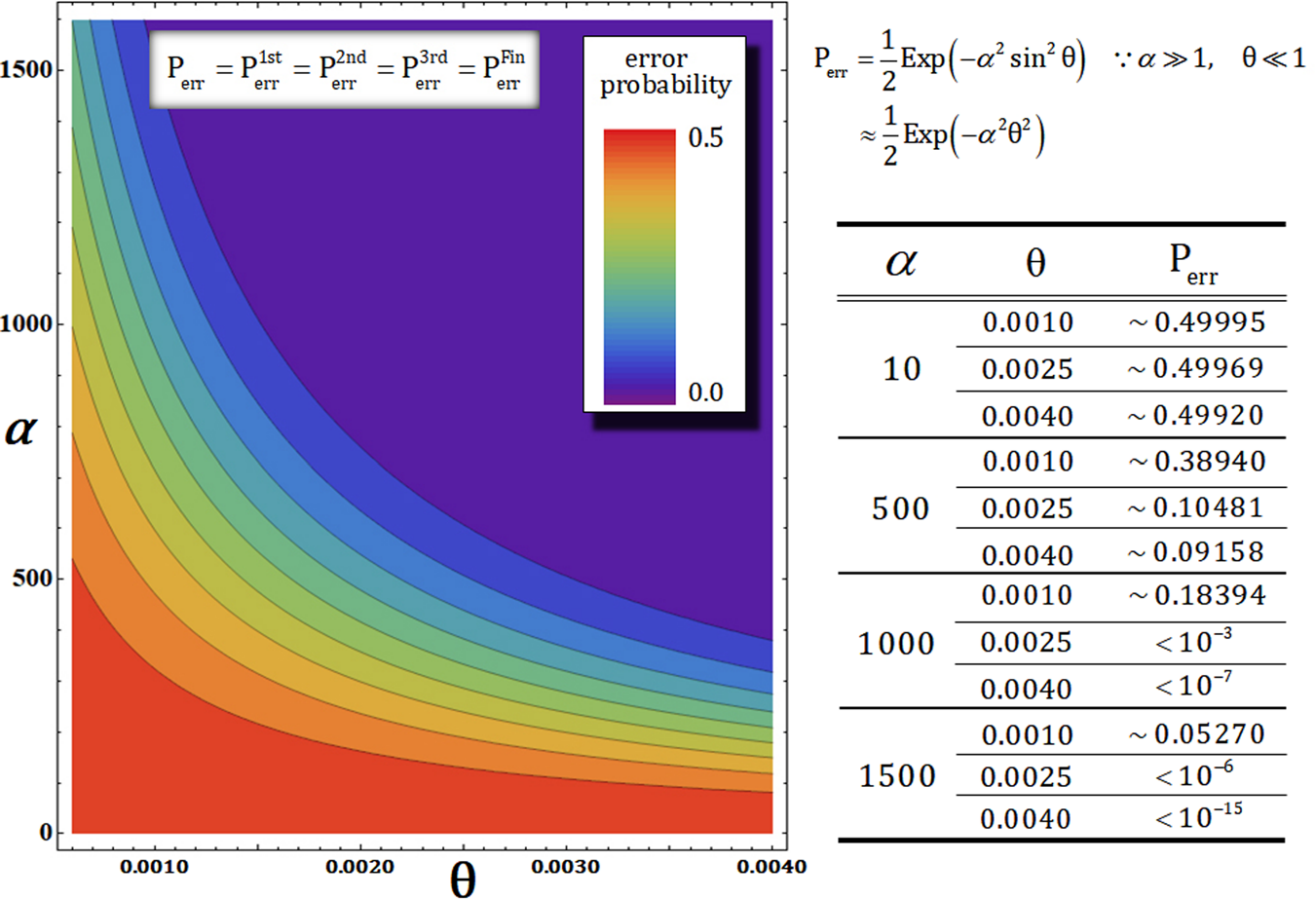
Graph shows the error probability (P_err_) according to the changing amplitude (*α*) of coherent state and magnitude (θ) of phase shift. Four nonlinearly optical gates (1st, 2nd, 3rd, and final) have the same error probability (${{\rm{P}}}_{{\rm{err}}}^{{\rm{1st}}}={{\rm{P}}}_{{\rm{err}}}^{{\rm{2nd}}}={{\rm{P}}}_{{\rm{err}}}^{{\rm{3rd}}}={{\rm{P}}}_{{\rm{err}}}^{{\rm{Fin}}}$), by PNR measurement. And the values of error probabilities calculated in terms of the parameters (*α* and θ) are listed on the table. Due to graph and table, the error probability is decreasing to 0 if we use the strong coherent state and the large phase shift.

Finally, in Fig. 6, we express the error probabilities of our gates in terms of the difference in the amplitude of coherent state and the magnitude of phase shift by XKNL.

The error probabilities of four nonlinearly optical gates (1st, 2nd, 3rd, and final) are identical to P_err_, as described in Fig. 6, due to the probability to measure ${|0\rangle }_{{\rm{P}}}^{{\rm{b}}}$ (no detection) in ${|\pm i\alpha \,\sin \,\theta \rangle }_{{\rm{P}}}^{{\rm{b}}}$ on path b. Figure 6 and the table (in Fig. 6) obviously represent that the error probability, P_err_, will be approaching 0 when both the amplitude (*α*) of coherent state and magnitude (θ) of phase shift increase (utilizing strong coherent state and large phase shift of XKNL). It is possible to efficiently produce the output states of nonlinearly optical gates by increasing the multiplying value (*αθ*) of parameters, *α* and θ of ${{\rm{P}}}_{{\rm{err}}}\approx ({e}^{-{\alpha }^{2}{{\rm{\theta }}}^{2}})/2$, in the ideal case (the assumption without decoherence effect). In the next section, to conveniently analyze the decoherence effect in gates (1st, 2nd, 3rd, and final), we take the value, ${{\rm{P}}}_{{\rm{err}}} < {10}^{-3}$, of error probability to fix the parameters (the amplitude of the coherent state and the magnitude of condition phase shift) as *αθ* = 2.5.

So far, we have designed a scheme to encode quantum information into three-photon decoherence-free states (single logical qubit information) using nonlinearly optical gates (XKNLs, qubus beams, and PNR measurement) and linearly optical devices (50/50 BSs, PBSs, single photon operators, and an arbitrary-BS) for the resistance against collective decoherence. However, due to the utilization of XKNLs in our scheme, the decoherence effect, which makes to evolve quantum pure state into a mixed state by photon loss and dephasing, occurs in nonlinearly optical gates (1st, 2nd, 3rd, and final) when experimentally implemented by our scheme in practical optical fibers^66,67^. Thus, we should analyze the nonlinearly optical gates using XKNLs, qubus beams, and PNR measurement under the decoherence effect (photon loss and dephasing)^55,56,62^.

## Analysis of Nonlinearly Optical Gates Using XKNLs, Qubus Beams, and PNR Measurement Under Decoherence Effect

In our scheme to encode quantum information into three-photon decoherence-free states (single logical qubit information), the nonlinearly optical gates (1st, 2nd, 3rd, and final) utilize XKNLs, qubus beams, and PNR measurement. However, when the nonlinearly optical gates are operated in optical fibers^55,56,60,62,63^, the decoherence effect (as distinct from the collective decoherence^23–25^) is induced by photon loss and dephasing (coherent parameters of photon-probe system), affecting the efficiency and performance of the nonlinearly optical gates^55,56,60,62,63^ according to photon loss (increasing the error probabilities ${{\rm{P}}}_{{\rm{err}}}^{{\rm{1st}}}={{\rm{P}}}_{{\rm{err}}}^{{\rm{2nd}}}={{\rm{P}}}_{{\rm{err}}}^{{\rm{3rd}}}={{\rm{P}}}_{{\rm{err}}}^{{\rm{Fin}}}$) and dephasing (decreasing the fidelities of output states ${|{\psi }_{{\rm{0}}}\rangle }_{{\rm{ABC}}}$, ${|{\psi }_{{\rm{1}}}\rangle }_{{\rm{ABC}}}$, ${|{\psi }_{{\rm{2}}}\rangle }_{{\rm{ABC}}}$, and ${|{\phi }_{{\rm{L}}1}^{^{\prime} }\rangle }_{{\rm{ABC}}}$ or ${|{\phi }_{{\rm{L1}}}\rangle }_{{\rm{ABC}}}$).

The decoherence effect in the Kerr medium can be induced by solving the master equation (description of open quantum system)^68^. Therefore, we should apply the analysis (solving the master equation)^55,56,62^ of the decoherence effect in the interaction of XKNLs to our nonlinearly optical gates via XKNLs, qubus beams, and PNR measurements to show the robustness against the decoherence effect.

14 $$\frac{{\rm{\partial }}\rho (t)}{{\rm{\partial }}t}=-\,\frac{i}{\hslash }[H,\,\rho ]+\gamma (a\rho {a}^{+}-\frac{1}{2}({a}^{+}a\rho +\rho {a}^{+}a)),\,\because \hat{J}=\gamma a\rho {a}^{+},\,\hat{L}=\frac{\gamma }{2}({a}^{+}a\rho +\rho {a}^{+}a),$$

where *γ*, *t* (=*θ*/*χ*), and *a*^+^(*a*) are the energy decay rate, the interaction time, and creation (annihilation) operator. And the solution of master equation, Eq. 14, is given as $\rho (t)=\exp [(\hat{J}+\hat{L})t]\rho (0)$. From the solution in Eq. 14, we can obtain the photon loss rate in the coherent state (probe beam) as $|{\Lambda }_{t}\alpha \rangle$ (Λ_*t*_ = *e*^−*γt*/2^) after Kerr medium. Also, the interaction time *t* of decoherence, ${\tilde{D}}_{t}$, and XKNL, ${\tilde{X}}_{t}$, can be taken as arbitrarily small time Δ*t* (=*t*/*N*) for a good approximation of analysis^55,56,62^. When the initial state is supposed as $|H\rangle \langle V|\otimes |\alpha \rangle \langle \alpha |$ (i.e. *H*: XKNL and *V*: no interaction), we can show the process model of the interaction of XKNL, ${\tilde{X}}_{t}$, and decoherence, ${\tilde{D}}_{t}$ (photon loss and dephasing)^55,56,62^ from Eq. 14 (the detailed description of this model: APPENDIX), as follows:

15 $$\begin{array}{ccc}{({\mathop{D}\limits^{ \sim }}_{{\rm{\Delta }}t}{\mathop{X}\limits^{ \sim }}_{{\rm{\Delta }}t})}^{N}|H\rangle \langle V|\otimes |\alpha \rangle \langle \alpha | & = & \exp [\,-\,{\alpha }^{2}(1-{e}^{-\gamma {\rm{\Delta }}t})\sum _{n=1}^{N}{e}^{-\gamma {\rm{\Delta }}t(n-1)}(1-{e}^{in{\rm{\Delta }}\theta })]\\  &  & \times |H\rangle \langle V|\otimes |{{\rm{\Lambda }}}_{t}\alpha {e}^{i\theta }\rangle \langle {{\rm{\Lambda }}}_{t}\alpha |,\end{array}$$

where ${\tilde{D}}_{t}{\tilde{X}}_{t}={({\tilde{D}}_{{\rm{\Delta }}t}{\tilde{X}}_{{\rm{\Delta }}t})}^{N}$, and *θ* = *χt* = *χN*Δ*t* = *N*Δ*θ* for Δ*t* (=*t*/*N*) and $\alpha \in \Re$. In Eq. 15, the coefficient of the right hand of the equation is called the coherent parameter, which can quantify the amount of decoherence effect by the degree of dephasing, as described in^55,56,62^ (APPENDIX). For the experimental implementation of the nonlinearly optical gates (1st, 2nd, 3rd, and final) in optical fibers, which require the length of about 3000 km to obtain the magnitude of phase shift, *θ* = *π*, of the XKNL^66,67^, we can analyze the performance and efficiency of the gates in pure silica-core fibers^67^ having the signal loss of 0.15 dB/km (*χ*/*γ* = 0.0303) under the decoherence effect by the process model^55,56,62^ of Eqs 14 and 15.

### 1st gate (photons A-B) under the decoherence effect

**Figure 7:**
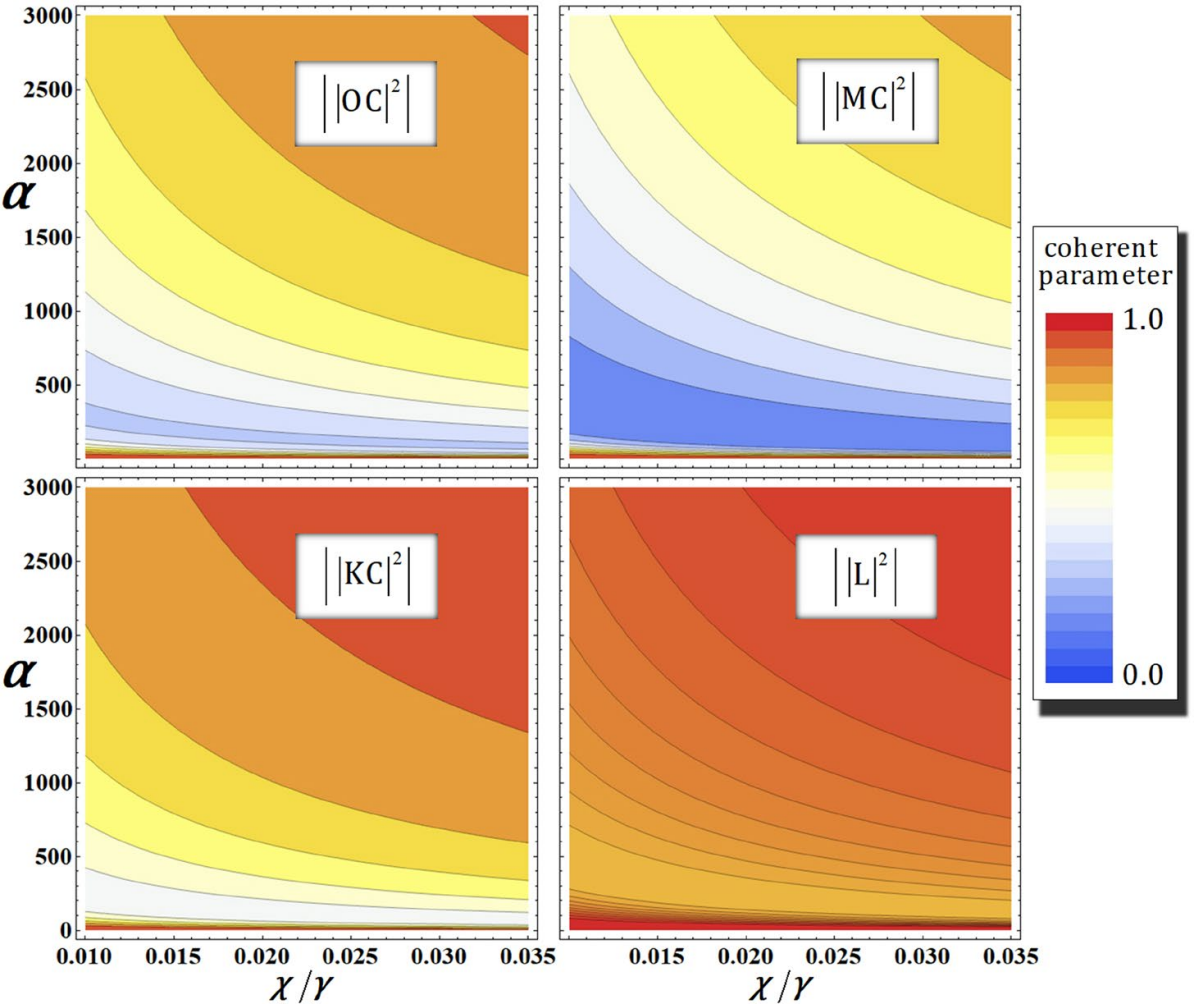
Graphs of the absolute values of the coherent parameters in the output state ${\rho }_{{\rm{AB}}}^{0}$ of the first gate in our scheme (single logical qubit information) with respect to the amplitude of the coherent state *α* and the signal loss *χ*/*γ* in optical fibers, where fixed ${{\rm{P}}}_{{\rm{err}}}^{{\rm{1st}}}={10}^{-3}(\alpha \theta =\alpha \chi t\approx 2.5)$ with *N* = 10^3^.

According to decoherence effect, we should consider the photon loss and dephasing coherent parameters in our analysis when the first gate is realized in optical fiber^67^. Thus, the output state ${|{\psi }_{0}^{^{\prime} }\rangle }_{{\rm{ABC}}}$ [photon (A and B)-probe system] of the first gate in Eq. 5 will evolve into the mixed state ${\rho }_{{\rm{AB}}}^{0}$, as follows:

16 $${\rho }_{{\rm{AB}}}^{0}=\frac{1}{4}(\begin{array}{cccc}1 & {|{\rm{CK}}|}^{2} & {|{\rm{L}}|}^{2} & {|0{\rm{C}}|}^{2}\\ {|{\rm{CK}}|}^{2} & 1 & {|0{\rm{C}}|}^{2} & {|{\rm{L}}|}^{2}\\ {|{\rm{L}}|}^{2} & {|0{\rm{C}}|}^{2} & 1 & {|{\rm{MC}}|}^{2}\\ {|0{\rm{C}}|}^{2} & {|{\rm{L}}|}^{2} & {|{\rm{MC}}|}^{2} & 1\end{array}),$$

where the bases of ${\rho }_{{\rm{AB}}}^{0}$ are ${|H\rangle }_{{\rm{A}}}^{1}{|H\rangle }_{{\rm{B}}}^{3}{|{{\rm{\Lambda }}}_{t}^{2}\alpha \rangle }_{{\rm{P}}}^{{\rm{a}}}{|0\rangle }_{{\rm{P}}}^{{\rm{b}}}$, ${|H\rangle }_{{\rm{A}}}^{2}{|H\rangle }_{{\rm{B}}}^{4}{|{{\rm{\Lambda }}}_{t}^{2}\alpha \rangle }_{{\rm{P}}}^{{\rm{a}}}{|0\rangle }_{{\rm{P}}}^{{\rm{b}}}$, ${|H\rangle }_{{\rm{A}}}^{1}{|H\rangle }_{{\rm{B}}}^{4}{|{{\rm{\Lambda }}}_{t}^{2}\alpha \,\cos \,{\rm{\theta }}\rangle }_{{\rm{P}}}^{{\rm{a}}}{|i{{\rm{\Lambda }}}_{t}^{2}\alpha \,\sin \,{\rm{\theta }}\rangle }_{{\rm{P}}}^{{\rm{b}}}$, and ${|H\rangle }_{{\rm{A}}}^{2}{|H\rangle }_{{\rm{B}}}^{3}{|{{\rm{\Lambda }}}_{t}^{2}\alpha \,\cos \,{\rm{\theta }}\rangle }_{{\rm{P}}}^{{\rm{a}}}{|-i{{\rm{\Lambda }}}_{t}^{2}\alpha \,\sin \,{\rm{\theta }}\rangle }_{{\rm{P}}}^{{\rm{b}}}$ (Λ_*t*_ = *e*^−*γt*/2^) from top to bottom and left to right. The coherent parameters (C, O, L, K, and M) can be calculated from Eq. 15 (APPENDIX), as follows:

17 $$\begin{array}{ccc}\,{\rm{C}} & = & \exp [\,-\,\frac{{\alpha }^{2}}{2}(1-{e}^{-\gamma {\rm{\Delta }}t})\sum _{n=1}^{N}{e}^{-\gamma {\rm{\Delta }}t(n-1)}(1-{e}^{in{\rm{\Delta }}\theta })],\\ \,{\rm{O}} & = & \exp [\,-\,\frac{{\alpha }^{2}}{2}{e}^{-\gamma t}(1-{e}^{-\gamma {\rm{\Delta }}t})(1-{e}^{i\theta })\sum _{n=1}^{N}{e}^{-\gamma {\rm{\Delta }}t(n-1)}],\\ \,{\rm{L}} & = & \exp [\,-\,\frac{{\alpha }^{2}}{2}{e}^{-\gamma t}(1-{e}^{-\gamma {\rm{\Delta }}t})\sum _{n=1}^{N}{e}^{-\gamma {\rm{\Delta }}t(n-1)}(1-{e}^{in{\rm{\Delta }}\theta })],\\ \,({\rm{M}}:+\,)\,{\rm{o}}{\rm{r}}\,({\rm{K}}:-\,) & = & \exp [\,-\,\frac{{\alpha }^{2}}{2}{e}^{-\gamma t}(1-{e}^{-\gamma {\rm{\Delta }}t})\sum _{n=1}^{N}{e}^{-\gamma {\rm{\Delta }}t(n-1)}(1-{e}^{i(\theta \pm n{\rm{\Delta }}\theta )})],\end{array}$$

where ${\tilde{D}}_{t}{\tilde{X}}_{t}={({\tilde{D}}_{{\rm{\Delta }}t}{\tilde{X}}_{{\rm{\Delta }}t})}^{N}$, *θ* = *χt* = *χN*Δ*t* = *NΔθ* for Δ*t* (=*t*/*N*) and $\alpha \in \Re$. These equations mean the coherent parameters (off-diagonal terms) in the output state ${\rho }_{{\rm{AB}}}^{0}$ (Eq. 16) of the first gate.In Fig. 7, we represent the absolute values of coherent parameters according to the amplitude, *α*, of the coherent state in the qubus beam and the signal loss rate, *χ*/*γ*, in optical fibers. Here, we fix the error probability as ${{\rm{P}}}_{{\rm{err}}}^{{\rm{1st}}}={10}^{-3}$ for *αθ* = *αχt* = 2.5 with *N* = 10^3^. Thus, when fixed ${{\rm{P}}}_{{\rm{err}}}^{{\rm{1st}}}={10}^{-3}$, we can parameterize the coherent parameters (Eq. 17) in the amplitude of the coherent state, *α*, and the signal loss rate, *χ*/*γ*, by *θ* = *χt* = 2.5/*α* and *γt* = 2.5*γ*/*χα* for Δ*t* = *t*/1000 and ${\rm{\Delta }}\theta =\theta /1000(\because N={10}^{3})$. As described in Fig. 7, we conclude that if the amplitude of the coherent state increases, then the output state ${\rho }_{{\rm{AB}}}^{0}$ does not evolve into the mixed state by increasing the absolute values of the coherent parameters to one whole signal loss. Therefore, we can obtain a reliable performance of the first gate (high fidelity) to employ the strong coherent state under the decoherence effect.

### 2nd gate (photons A-B-C) under the decoherence effect

**Figure 8:**
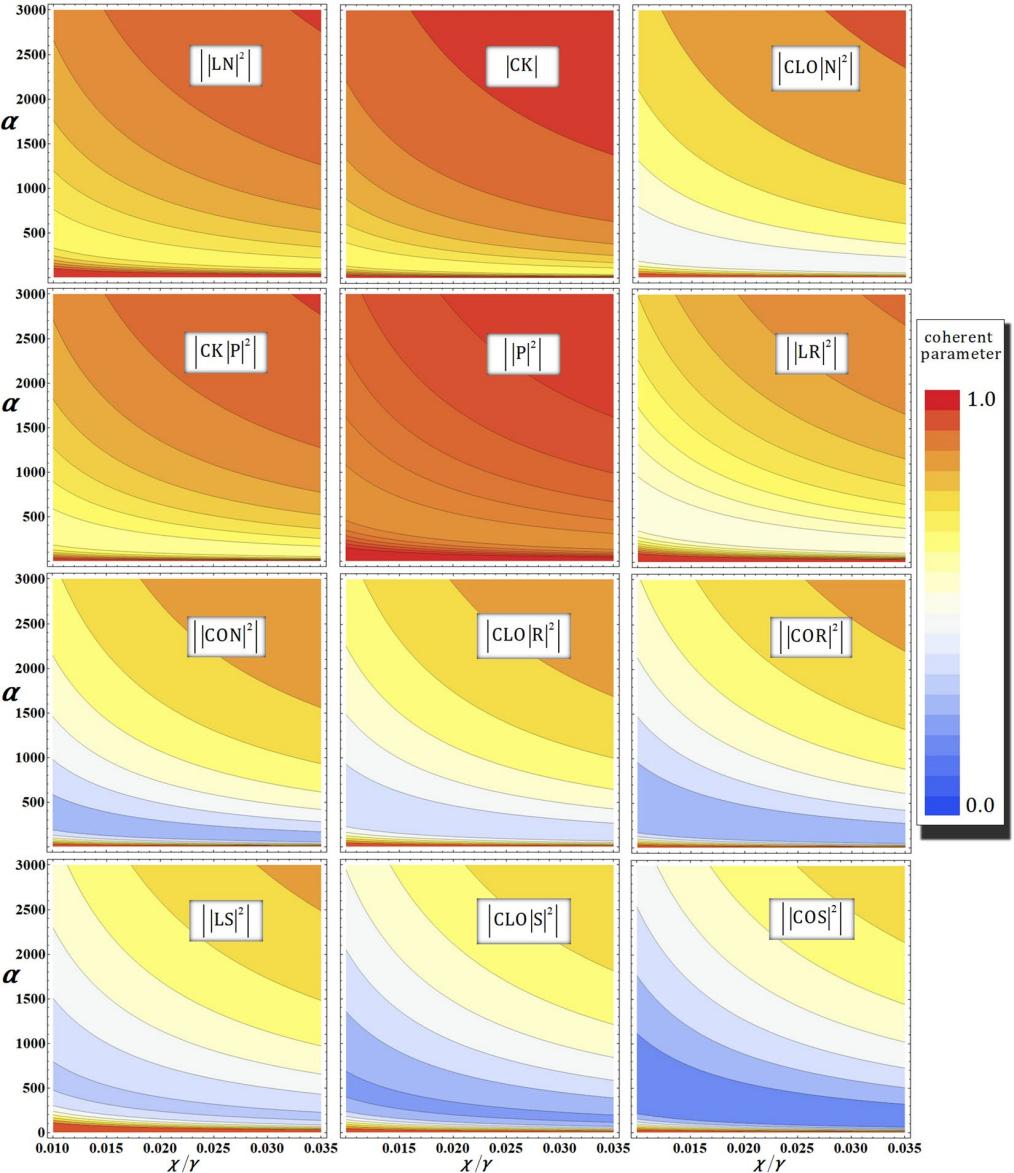
Graphs of the absolute values of the coherent parameters in the output state ${\rho }_{{\rm{ABC}}}^{1}$ of the second gate in our scheme (single logical qubit information) according to the amplitude of the coherent state *α* and the signal loss *χ*/*γ* in optical fibers, where fixed ${{\rm{P}}}_{{\rm{err}}}^{{\rm{2nd}}}={10}^{-3}(\alpha \theta =\alpha \chi t\approx 2.5)$ with *N* = 10^3^.

For the analysis of photon loss and dephasing coherent parameters^55,56,62^, the output state ${|{\psi ^{\prime} }_{{\rm{1}}}\rangle }_{{\rm{ABC}}}$ [photon (A, B, and C)-probe system] of the second gate in Eq. 7 should be revised to the density operator ${\rho }_{{\rm{ABC}}}^{1}$, as follows:

18 $${\rho }_{{\rm{ABC}}}^{1}=\frac{1}{8}(\begin{array}{cccccccc}{\rm{1}} & {|{\rm{L}}{\rm{N}}|}^{{\rm{2}}} & {{\rm{C}}}^{{\rm{\ast }}}{{\rm{K}}}^{{\rm{\ast }}} & {{\rm{C}}}^{{\rm{\ast }}}{\rm{L}}{{\rm{O}}}^{{\rm{\ast }}}{|{\rm{N}}|}^{{\rm{2}}} & {|{\rm{P}}|}^{{\rm{2}}} & {{\rm{C}}}^{\ast }{K}^{{\rm{\ast }}}{|{\rm{P}}|}^{{\rm{2}}} & {|{\rm{L}}{\rm{R}}|}^{{\rm{2}}} & {{\rm{C}}}^{{\rm{\ast }}}{{\rm{L}}}_{{\rm{2}}}{{\rm{O}}}^{{\rm{\ast }}}{|{\rm{R}}|}^{{\rm{2}}}\\ {|{\rm{L}}{\rm{N}}|}^{{\rm{2}}} & {\rm{1}} & {{\rm{C}}}^{{\rm{\ast }}}{\rm{L}}{{\rm{O}}}^{{\rm{\ast }}}{|{\rm{N}}|}^{{\rm{2}}} & {{\rm{C}}}^{{\rm{\ast }}}{{\rm{K}}}^{{\rm{\ast }}} & {|{\rm{L}}{\rm{R}}|}^{{\rm{2}}} & {{\rm{C}}}^{{\rm{\ast }}}{\rm{L}}{{\rm{O}}}^{{\rm{\ast }}}{|{\rm{R}}|}^{{\rm{2}}} & {|{\rm{P}}|}^{{\rm{2}}} & {{\rm{C}}}^{{\rm{\ast }}}{{\rm{K}}}^{{\rm{\ast }}}{|{\rm{P}}|}^{{\rm{2}}}\\ {\rm{C}}{\rm{K}} & {\rm{C}}{{\rm{L}}}^{{\rm{\ast }}}{\rm{O}}{|{\rm{N}}|}^{{\rm{2}}} & {\rm{1}} & {|{\rm{C}}{\rm{O}}{\rm{N}}|}^{{\rm{2}}} & {\rm{C}}{\rm{K}}{|{\rm{P}}|}^{{\rm{2}}} & {|{\rm{P}}|}^{{\rm{2}}} & {\rm{C}}{{\rm{L}}}^{{\rm{\ast }}}{\rm{O}}{|{\rm{R}}|}^{{\rm{2}}} & {|{\rm{C}}{\rm{O}}{\rm{R}}|}^{{\rm{2}}}\\ {\rm{C}}{{\rm{L}}}^{{\rm{\ast }}}{\rm{O}}{|{\rm{N}}|}^{{\rm{2}}} & {\rm{C}}{\rm{K}} & {|{\rm{C}}{\rm{O}}{\rm{N}}|}^{{\rm{2}}} & {\rm{1}} & {\rm{C}}{{\rm{L}}}^{{\rm{\ast }}}{\rm{O}}{|{\rm{R}}|}^{{\rm{2}}} & {|{\rm{C}}{\rm{O}}{\rm{R}}|}^{{\rm{2}}} & {\rm{C}}{\rm{K}}{|{\rm{P}}|}^{{\rm{2}}} & {|{\rm{P}}|}^{{\rm{2}}}\\ {|{\rm{P}}|}^{{\rm{2}}} & {|{\rm{L}}{\rm{R}}|}^{{\rm{2}}} & {{\rm{C}}}^{{\rm{\ast }}}{{\rm{K}}}^{{\rm{\ast }}}{|{\rm{P}}|}^{{\rm{2}}} & {{\rm{C}}}^{{\rm{\ast }}}{\rm{L}}{{\rm{O}}}^{{\rm{\ast }}}{|{\rm{R}}|}^{{\rm{2}}} & {\rm{1}} & {{\rm{C}}}^{{\rm{\ast }}}{{\rm{K}}}^{{\rm{\ast }}} & {|{\rm{L}}{\rm{S}}|}^{{\rm{2}}} & {{\rm{C}}}^{{\rm{\ast }}}{\rm{L}}{{\rm{O}}}^{{\rm{\ast }}}{|{\rm{S}}|}^{{\rm{2}}}\\ {\rm{C}}{\rm{K}}{|{\rm{P}}|}^{{\rm{2}}} & {\rm{C}}{{\rm{L}}}^{{\rm{\ast }}}{\rm{O}}{|{\rm{R}}|}^{{\rm{2}}} & {|{\rm{P}}|}^{{\rm{2}}} & {|{\rm{C}}{\rm{O}}{\rm{R}}|}^{{\rm{2}}} & {\rm{C}}{\rm{K}} & {\rm{1}} & {\rm{C}}{{\rm{L}}}^{{\rm{\ast }}}{\rm{O}}{|{\rm{S}}|}^{{\rm{2}}} & {|{\rm{C}}{\rm{O}}{\rm{S}}|}^{{\rm{2}}}\\ {|{\rm{L}}{\rm{R}}|}^{{\rm{2}}} & {|{\rm{P}}|}^{{\rm{2}}} & {{\rm{C}}}^{{\rm{\ast }}}{\rm{L}}{{\rm{O}}}^{{\rm{\ast }}}{|{\rm{R}}|}^{{\rm{2}}} & {{\rm{C}}}^{{\rm{\ast }}}{{\rm{K}}}^{{\rm{\ast }}}{|{\rm{P}}|}^{{\rm{2}}} & {|{\rm{L}}{\rm{S}}|}^{{\rm{2}}} & {{\rm{C}}}^{{\rm{\ast }}}{\rm{L}}{{\rm{O}}}^{{\rm{\ast }}}{|{\rm{S}}|}^{{\rm{2}}} & {\rm{1}} & {{\rm{C}}}^{{\rm{\ast }}}{{\rm{K}}}^{{\rm{\ast }}}\\ {\rm{C}}{{\rm{L}}}^{{\rm{\ast }}}{\rm{O}}{|{\rm{R}}|}^{{\rm{2}}} & {\rm{C}}{\rm{K}}{|{\rm{P}}|}^{{\rm{2}}} & {|{\rm{C}}{\rm{O}}{\rm{R}}|}^{{\rm{2}}} & {|{\rm{P}}|}^{{\rm{2}}} & {\rm{C}}{{\rm{L}}}^{{\rm{\ast }}}{\rm{O}}{|{\rm{S}}|}^{{\rm{2}}} & {|{\rm{C}}{\rm{O}}{\rm{S}}|}^{{\rm{2}}} & {\rm{C}}{\rm{K}} & {\rm{1}}\end{array}),$$

where the bases of ${\rho }_{{\rm{AB}}}^{1}$ are ${|H\rangle }_{{\rm{A}}}^{1}{|H\rangle }_{{\rm{B}}}^{3}{|V\rangle }_{{\rm{C}}}^{5}{|{{\rm{\Lambda }}}_{t}^{3}\alpha \rangle }_{{\rm{P}}}^{{\rm{a}}}{|0\rangle }_{{\rm{P}}}^{{\rm{b}}}$, ${|H\rangle }_{{\rm{A}}}^{1}{|V\rangle }_{{\rm{B}}}^{3}{|H\rangle }_{{\rm{C}}}^{6}{|{{\rm{\Lambda }}}_{t}^{3}\alpha \rangle }_{{\rm{P}}}^{{\rm{a}}}{|0\rangle }_{{\rm{P}}}^{{\rm{b}}}$, ${|H\rangle }_{{\rm{A}}}^{2}{|H\rangle }_{{\rm{B}}}^{4}{|V\rangle }_{{\rm{C}}}^{5}{|{{\rm{\Lambda }}}_{t}^{3}\alpha \rangle }_{{\rm{P}}}^{{\rm{a}}}{|0\rangle }_{{\rm{P}}}^{{\rm{b}}}$, ${|V\rangle }_{{\rm{A}}}^{2}{|H\rangle }_{{\rm{B}}}^{4}{|H\rangle }_{{\rm{C}}}^{6}{|{{\rm{\Lambda }}}_{t}^{3}\alpha \rangle }_{{\rm{P}}}^{{\rm{a}}}{|0\rangle }_{{\rm{P}}}^{{\rm{b}}}$,${|H\rangle }_{{\rm{A}}}^{1}{|H\rangle }_{{\rm{B}}}^{3}{|H\rangle }_{{\rm{C}}}^{6}{|{{\rm{\Lambda }}}_{t}^{3}\alpha \,\cos \,{\rm{\theta }}\rangle }_{{\rm{P}}}^{{\rm{a}}}{|i{{\rm{\Lambda }}}_{t}^{3}\alpha \,\sin \,{\rm{\theta }}\rangle }_{{\rm{P}}}^{{\rm{b}}}$,${|H\rangle }_{{\rm{A}}}^{2}{|H\rangle }_{{\rm{B}}}^{4}{|H\rangle }_{{\rm{C}}}^{6}{|{{\rm{\Lambda }}}_{t}^{3}\alpha \,\cos \,{\rm{\theta }}\rangle }_{{\rm{P}}}^{{\rm{a}}}{|i{{\rm{\Lambda }}}_{t}^{3}\alpha \,\sin \,{\rm{\theta }}\rangle }_{{\rm{P}}}^{{\rm{b}}}$, ${|H\rangle }_{{\rm{A}}}^{1}$, ${|V\rangle }_{{\rm{B}}}^{3}{|V\rangle }_{{\rm{C}}}^{5}{|{{\rm{\Lambda }}}_{t}^{3}\alpha \,\cos \,{\rm{\theta }}\rangle }_{{\rm{P}}}^{{\rm{a}}}{|-i{{\rm{\Lambda }}}_{t}^{3}\alpha \,\sin \,{\rm{\theta }}\rangle }_{{\rm{P}}}^{{\rm{b}}}$ and ${|V\rangle }_{{\rm{A}}}^{2}{|H\rangle }_{{\rm{B}}}^{4}{|V\rangle }_{{\rm{C}}}^{6}{|{{\rm{\Lambda }}}_{t}^{3}\alpha \,\cos \,{\rm{\theta }}\rangle }_{{\rm{P}}}^{{\rm{a}}}{|-i{{\rm{\Lambda }}}_{t}^{3}\alpha \,\sin \,{\rm{\theta }}\rangle }_{{\rm{P}}}^{{\rm{b}}}$ (Λ_*t*_ = *e*^−*γt*/2^) from top to bottom and left to right. The coherent parameters (R, *P*, *S*, and *N*) can be calculated from Eq. 15 (APPENDIX), as follows:

19 $$\begin{array}{ccc}\,{\rm{R}} & = & \exp [\,-\,\frac{{\alpha }^{2}}{2}{e}^{-2\gamma t}(1-{e}^{-\gamma {\rm{\Delta }}t})(1-{e}^{i\theta })\sum _{n=1}^{N}{e}^{-\gamma {\rm{\Delta }}t(n-1)}],\\ \,{\rm{P}} & = & \exp [\,-\,\frac{{\alpha }^{2}}{2}{e}^{-2\gamma t}(1-{e}^{-\gamma {\rm{\Delta }}t})\sum _{n=1}^{N}{e}^{-\gamma {\rm{\Delta }}t(n-1)}(1-{e}^{in{\rm{\Delta }}\theta })],\\ \,({\rm{S}}:+\,)\,{\rm{o}}{\rm{r}}\,({\rm{N}}:-\,) & = & \exp [\,-\,\frac{{\alpha }^{2}}{2}{e}^{-2\gamma t}(1-{e}^{-\gamma {\rm{\Delta }}t})\sum _{n=1}^{N}{e}^{-\gamma {\rm{\Delta }}t(n-1)}(1-{e}^{i(\theta \pm n{\rm{\Delta }}\theta )})],\end{array}$$

where ${\tilde{D}}_{t}{\tilde{X}}_{t}={({\tilde{D}}_{{\rm{\Delta }}t}{\tilde{X}}_{{\rm{\Delta }}t})}^{N}$, *θ* = *χt* = *χN*Δ*t* = *N*Δ*θ* for,Δ*t* (=*t*/*N*) and $\alpha \in \Re$. Also, the remains of coherent parameters (C, O, L, and K) of ${\rho }_{{\rm{ABC}}}^{1}$ are written in Eq. 17. Figure 8 shows the absolute values of the coherent parameters for *α* and *χ*/*γ* in optical fibers. In the second gate, we take the identical condition of the first gate, as ${{\rm{P}}}_{{\rm{err}}}^{{\rm{2nd}}}={10}^{-3}$ for *αθ* = *αχt* = 2.5 with *N* = 10^3^. As described in Fig. 8, when we employ the strong coherent state (increasing *α*), the absolute values of the coherent parameters in ${\rho }_{{\rm{ABC}}}^{1}$ approach 1 for the reliable performance of the second gate (remaining the output state as quantum pure state) under the decoherence effect.

### 3rd gate (photons A-B) and final gate (photon B) under the decoherence effect

Two (3rd and final) gates can be considered as being several types of merging path gates. Thus, by the process model^55,56,62^ of Eqs 14 and 15 (APPENDIX), the coherent parameters of two output states (${\rho }_{{\rm{ABC}}}^{2}$ and ${\rho }_{{\rm{ABC}}}^{{\rm{E}}}$) are the same (but the bases of the two density matrices are different), and the two output states (${\rho }_{{\rm{ABC}}}^{2}$ and ${\rho }_{{\rm{ABC}}}^{{\rm{E}}}$) can be expressed from Eqs 9 and 12, as follows:

20 $$\begin{array}{c}{\rho }_{{\rm{A}}{\rm{B}}{\rm{C}}}^{2}={\rho }_{{\rm{A}}{\rm{B}}{\rm{C}}}^{{\rm{E}}}=\frac{1}{2}(\begin{array}{cc}1 & {|{\rm{C}}|}^{2}\\ {|{\rm{C}}|}^{2} & 1\end{array}),\\ \begin{array}{cc}{\rm{b}}{\rm{a}}{\rm{s}}{\rm{e}}{\rm{s}}\,of\,{\rho }_{{\rm{A}}{\rm{B}}{\rm{C}}}^{2}: & \{\frac{1}{2}({|{0}_{{\rm{L}}1}\rangle }^{145}+\sqrt{3}{|{1}_{{\rm{L}}1}\rangle }^{135})\otimes {|{{\rm{\Lambda }}}_{t}\alpha \rangle }_{{\rm{P}}}^{{\rm{a}}}{|0\rangle }_{{\rm{P}}}^{{\rm{b}}},\\  & \frac{1}{2}({|{0}_{{\rm{L}}1}\rangle }^{235}+\sqrt{3}{|{1}_{{\rm{L}}1}\rangle }^{245})\otimes {|{{\rm{\Lambda }}}_{t}\alpha \,\cos \,\theta \rangle }_{{\rm{P}}}^{{\rm{a}}}{|-i{{\rm{\Lambda }}}_{t}\alpha \,\sin \,\theta \rangle }_{{\rm{P}}}^{{\rm{b}}}\},\\ {\rm{b}}{\rm{a}}{\rm{s}}{\rm{e}}{\rm{s}}\,of\,{\rho }_{{\rm{A}}{\rm{B}}{\rm{C}}}^{2}: & \{({\alpha }_{1}{|{0}_{{\rm{L}}1}\rangle }^{135}+{\beta }_{1}{|{1}_{{\rm{L}}1}\rangle }^{135})\otimes {|{{\rm{\Lambda }}}_{t}\alpha \rangle }_{{\rm{P}}}^{{\rm{a}}}{|0\rangle }_{{\rm{P}}}^{{\rm{b}}},\\  & ({\alpha }_{2}{|{0}_{{\rm{L}}1}\rangle }^{145}+{\beta }_{2}{|{1}_{{\rm{L}}1}\rangle }^{145})\otimes {|{{\rm{\Lambda }}}_{t}\alpha \,\cos \,\theta \rangle }_{{\rm{P}}}^{{\rm{a}}}{|-i{{\rm{\Lambda }}}_{t}\alpha \,\sin \,\theta \rangle }_{{\rm{P}}}^{{\rm{b}}}\},\end{array}\end{array}$$

where the coherent parameter (*C*) is given by Eq. 17, and photon loss rate as Λ_*t*_ = *e*^−*γt*/2^.

**Figure 9:**
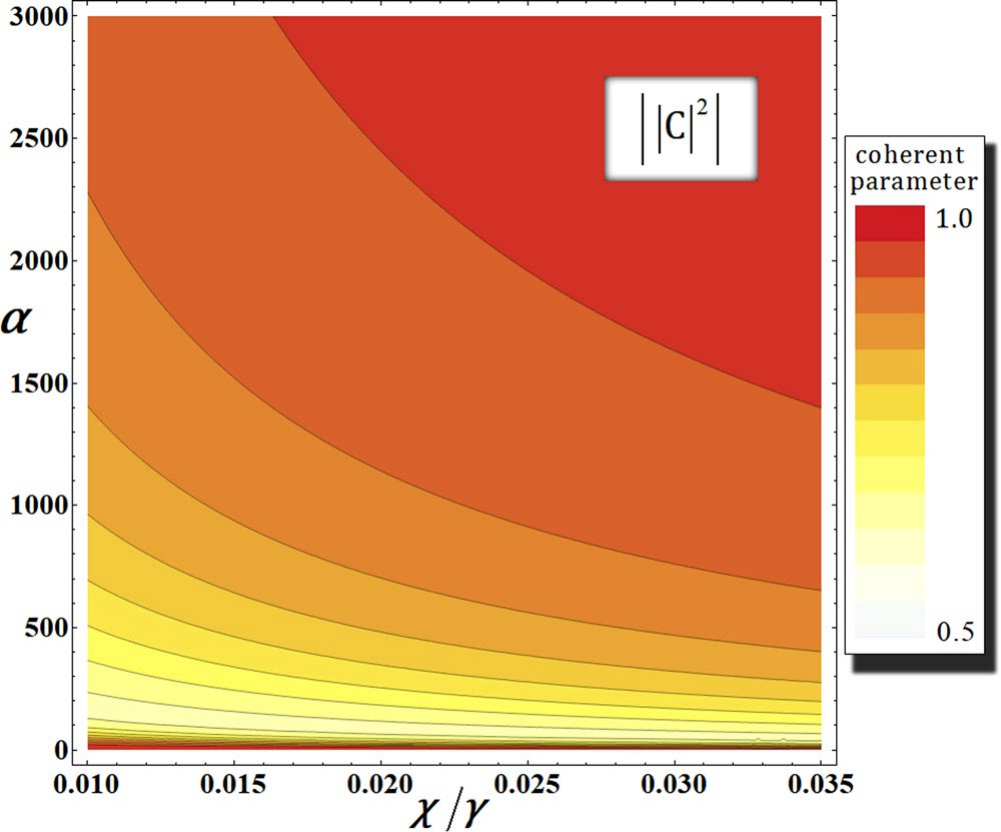
Graph of the absolute value of the coherent parameter in output states ${\rho }_{{\rm{ABC}}}^{2}$ (third gate) and ${\rho }_{{\rm{ABC}}}^{{\rm{E}}}$ (final gate) in our scheme (single logical qubit information) with regard to the amplitude of the coherent state *α* and the signal loss *χ*/*γ* in optical fibers, where fixed ${{\rm{P}}}_{{\rm{err}}}^{{\rm{3rd}}}={{\rm{P}}}_{{\rm{err}}}^{{\rm{Fin}}}={10}^{-3}(\alpha \theta =\alpha \chi t\approx 2.5)$ with *N* = 10^3^.

When increasing the amplitude of the coherent state, the absolute value of the coherent parameter, $|{|{\rm{C}}|}^{2}|$, can be maintained to one in the requirement as ${{\rm{P}}}_{{\rm{err}}}^{{\rm{3rd}}}={{\rm{P}}}_{{\rm{err}}}^{{\rm{Fin}}}={10}^{-3}$ for *αθ* = *αχt* = 2.5 with *N* = 10^3^, as shown in Fig. 9. Consequently, if we increase the amplitude of coherent state for the operations of nonlinearly optical gates (1st, 2nd, 3rd, and final), the reliable performance of those gates can be obtained to retain the absolute values of coherent parameters as one where fixed the error probabilities (≈10^−3^) and the experimental parameters as *αθ* = *αχt* = 2.5 with *N* = 10^3^. This means to prevent the output states (${\rho }_{{\rm{AB}}}^{{\rm{0}}}$, ${\rho }_{{\rm{ABC}}}^{{\rm{1}}}$, ${\rho }_{{\rm{ABC}}}^{2}$, and ${\rho }_{{\rm{ABC}}}^{{\rm{E}}}$) from evolving into mixed states by dephasing the coherent parameters under the decoherence effect.

Furthermore, the error probabilities (${{\rm{P}}}_{{\rm{err}}}^{{\rm{1st}}}$, ${{\rm{P}}}_{{\rm{err}}}^{{\rm{2nd}}}$, ${{\rm{P}}}_{{\rm{err}}}^{{\rm{3rd}}}$, and ${{\rm{P}}}_{{\rm{err}}}^{{\rm{Fin}}}$) affected by photon loss can be calculated to confirm the efficiency of nonlinearly optical gates, as follows:

21 $$\begin{array}{rcl}{{\rm{P}}}_{{\rm{err}}}^{{\rm{1st}}} & \approx  & \exp [\,-\,{{\rm{\Lambda }}}_{t}^{4}\cdot {(\alpha \theta )}^{2}]/2=\exp [\,-\,{e}^{-2\cdot (2.5)/(0.0303)\cdot \alpha }\cdot {(2.5)}^{2}]/2,\\ {{\rm{P}}}_{{\rm{err}}}^{{\rm{2nd}}} & \approx  & \exp [\,-\,{{\rm{\Lambda }}}_{t}^{6}\cdot {(\alpha \theta )}^{2}]/2=\exp [\,-\,{e}^{-3\cdot (2.5)/(0.0303)\cdot \alpha }\cdot {(2.5)}^{2}]/2,\\ {{\rm{P}}}_{{\rm{err}}}^{{\rm{3rd}}} & = & {{\rm{P}}}_{{\rm{err}}}^{{\rm{Fin}}}\approx \exp [\,-\,{{\rm{\Lambda }}}_{t}^{2}\cdot {(\alpha \theta )}^{2}]/2=\exp [\,-\,{e}^{-2.5/(0.0303)\cdot \alpha }\cdot {(2.5)}^{2}]/2,\end{array}$$

where *αθ* = *αχt* = 2.5, Λ_*t*_ = *e*^−*γt*/2^, and *γt* = 2.5/(0.0303) · *α* in the pure silica-core fibers having the signal loss of 0.15 dB/km (*χ*/*γ* = 0.0303)^67^.

**Figure 10:**
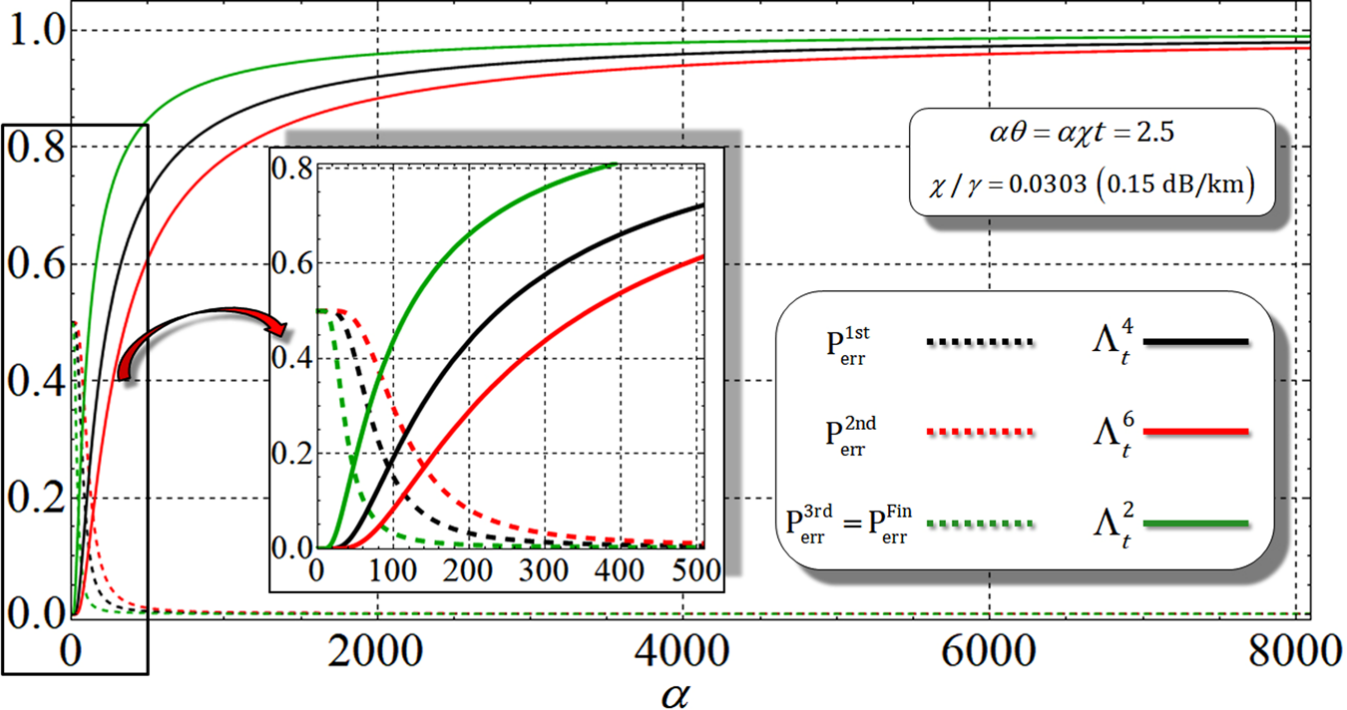
Graph represents the error probabilities (${{\rm{P}}}_{{\rm{err}}}^{{\rm{1st}}}$,${{\rm{P}}}_{{\rm{err}}}^{{\rm{2nd}}}$,${{\rm{P}}}_{{\rm{err}}}^{{\rm{3rd}}}$, and ${{\rm{P}}}_{{\rm{err}}}^{{\rm{Fin}}}$) and photon loss rates (${{\rm{\Lambda }}}_{t}^{4}$, ${{\rm{\Lambda }}}_{t}^{6}$, and ${{\rm{\Lambda }}}_{t}^{2}$) in nonlinearly optical gates, according to the amplitude of coherent state *α* with *αθ* = 2.5 in optical fibers having 0.15 dB/km (*χ*/*γ* = 0.0303). In the range of weak ($\alpha :300\, \sim \,600$) and strong (*α* > 600) coherent states, the error probabilities are maintained to nearly zero when we consider only photon loss under the decoherence effect. The range of the weak coherent state (0 < *α* < 500) is expressed in the small graph.

In Fig. 10, we can show that the error probabilities (${{\rm{P}}}_{{\rm{err}}}^{{\rm{1st}}}$, ${{\rm{P}}}_{{\rm{err}}}^{{\rm{2nd}}}$, ${{\rm{P}}}_{{\rm{err}}}^{{\rm{3rd}}}$, and ${{\rm{P}}}_{{\rm{err}}}^{{\rm{Fin}}}$) of nonlinearly optical gates converge to zero when increasing the amplitude of the coherent state, because the photon loss rates approach one (${{\rm{\Lambda }}}_{t}^{4},\,{{\rm{\Lambda }}}_{t}^{6},\,{{\rm{\Lambda }}}_{t}^{2}\to 1$) under the decoherence effect in optical fibers^67^. Even if the amplitude of the coherent state is small, we can also obtain low error probabilities, as described in the small graph of Fig. 10. For example, when *α* = 400, we can acquire ${{\rm{P}}}_{{\rm{err}}}^{{\rm{1st}}}\approx {{\rm{P}}}_{{\rm{err}}}^{{\rm{3rd}}}={{\rm{P}}}_{{\rm{err}}}^{{\rm{Fin}}} < {10}^{-2}$ and ${{\rm{P}}}_{{\rm{err}}}^{{\rm{2nd}}} < {10}^{-1}$ from Eq. 21, by ${{\rm{\Lambda }}}_{t}^{4}\approx 0.6$, ${{\rm{\Lambda }}}_{t}^{6}\approx 0.5$, and ${{\rm{\Lambda }}}_{t}^{2}\approx 0.8$. Namely, if we consider only photon loss induced by the decoherence effect in optical fibers, the high efficiency of nonlinearly optical gates from the error probabilities can be acquired by both the weak and the strong amplitude of the coherent state.

However, as mentioned above, photon loss and dephasing occur simultaneously when operating nonlinearly optical gates in optical fibers, and we could establish the effects from the process model in Eqs 14 and 15^55,56,62^.

**Figure 11:**
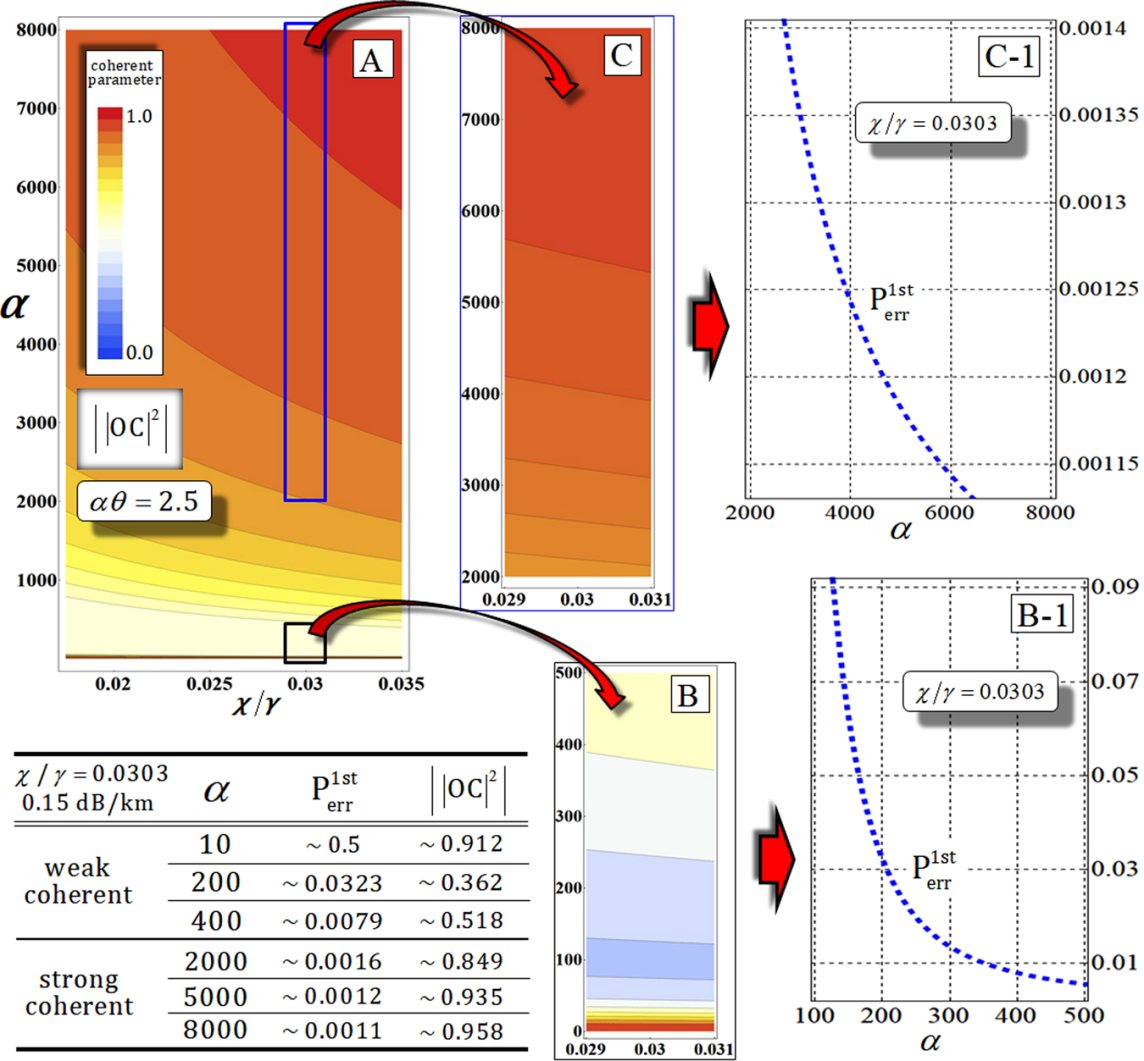
For the quantification of dephasing, plot (**A**) represents an absolute value, $|{|{\rm{OC}}|}^{2}|$, of the coherent terms in ${\rho }_{{\rm{AB}}}^{0}$ of the first gate, and plots (**B**) and (**C**) show the absolute value of the coherent parameter in weak (**B**) and strong (**C**) amplitudes of the coherent state for *αθ* = 2.5 (fixed ${{\rm{P}}}_{{\rm{err}}}^{{\rm{1st}}}={10}^{-3}$) and *N* = 10^3^, regarding the signal loss *χ*/*γ* in optical fibers. Also, we can recalculate the error probability of the first gate for photon loss with dephasing and *χ*/*γ* = 0.0303 in the range of the weak (B-1) and strong (C-1) amplitude of the coherent state. In the weak and strong ranges of the coherent state (probe beam), the error probability and the absolute value of coherent parameter are listed in the table, when photon loss and dephasing are simultaneously applied to the first gate.

Figure 11 shows a single example (the first gate) to simultaneously take into account photon loss and dephasing by our analysis (the process model of the decoherence effect, Eqs 14 and 15) in optical fibers. When *α* > 400 in the weak range of the coherent state, it is possible to obtain the low error probability (<10^−2^) [from (B-1) in Fig. 11] as strong range [from (C-1) in Fig. 11], as listed in the table in Fig. 11. While *α* ≪ 400 (extremely weak range), the error probability increases (decreasing the efficiency of the first gate). But, in view of the performance of the gate, due to dephasing the coherent parameter, the absolute value, $|{|OC|}^{2}|$, of the coherent parameter (off-diagonal term in ${\rho }_{{\rm{AB}}}^{0}$) will decrease (evolving to mixed state) in the weak range, as described in the table in Fig. 11. This obviously means that we can acquire only a single merit of high efficiency (low error probability against photon loss) or high performance (approaching the absolute value of the coherent parameter to one against dephasing) in the weak range of amplitude of the coherent state. Fortunately, both high efficiency and high performance of the nonlinearly optical gate can be achieved by using the strong amplitude of the coherent state under the decoherence effect (photon loss and dephasing), as described in Fig. 11. Consequently, we can conclude that the nonlinearly optical gates (1st, 2nd, 3rd, and final), using XKNL, qubus beams, and PNR measurement can be robust against the decoherence effect to increase the coherent state (probe beam) for high efficiency (low error probability) and reliable performance (high fidelity) by our analysis.

## Discussion and Conclusion

Recently, for the feasible XKNLs, researchers have variously proposed the experimental technologies in XKNLs with low error rate and high fidelity. The physical systems can realize to obtain the sufficient large strength of XKNL, such as electromagnetically induced transparency (EIT)^69,70^, circuit electromechanics^71^, an artificial atom^72^, and three-dimensional circuit quantum electrodynamic architecture^73^. Also, to acquire the strong phase shift for the photonic phase gate, Friedler *et al*.^74^ showed the large nonlinear interactions between ultraslow-light pulses or two stopped light pulses^75^, which can be realized in the regime of EIT. However, in the traveling fields described by localized single-photon pulse, the large phase shifts are impossible by large absorption losses^76^. Fortunately, there exist nontrivial problems to overcome at a theoretical level (utilizing both EIT and long-range interaction) in order to exploit weak XKNL^70^. Thus, high fidelities, nonzero conditional phases, and high photon numbers are compatible under certain conditions in He *et al*.^77^. Furthermore, for the reliable quantum information processing, researches have improved the capability of detection from the increase of the absorption^78,79^, and proposed the method of utilizing the positive-operator-value measurement elements^5,62^ for the reliable implementation of PNR measurement.

Therefore, in Sec. 2, we presented a scheme to encode quantum information (arbitrary information) into three-photon decoherence-free states (singe logical quantum information) via nonlinearly optical gates (1st, 2nd, 3rd, and final), which employ XKNLs, qubus beams, and PNR measurement, and linearly optical devices for immunity against collective decoherence. Then, for the high efficiency and reliable performance of the nonlinearly optical gates against photon loss and dephasing induced by the decoherence effect in optical fibers, we demonstrated the utilization of the strong amplitude of the coherent state (probe beam) by our analysis (Sec. 3) when operating nonlinearly optical gates (1st, 2nd, 3rd, and final) using XKNLs, qubus beams, and PNR measurement. Therefore, the advantages of our scheme are as follows:

1. To prevent quantum information transferred or operated in quantum information processing against collective decoherence between systems and the environment^23–25^, our scheme was designed to encode quantum information into three-photon decoherence-free states (logical qubits) using experimentally feasible nonlinear (XKNLs) and linear optics.
  The nonlinearly optical gates via XKNLs are critical components for generation of the three-photon decoherence-free states (logical qubits) and encoding quantum information in our scheme. Thus, for the robustness of the nonlinearly optical gates against decoherence effect, we also analyzed photon loss and dephasing^55,56,62^ in optical fibers, besides collective decoherence^23–25^.
2. We showed that the nonlinearly optical gates designed by XKNLs, qubus beams, and PNR measurement should employ the strong amplitude of the coherent state to acquire the high efficiency (low error probability) and reliable performance (high fidelity) by our analysis due to the process model^55,56,62^ of the master equation^68^ because of simultaneous occurrence of photon loss and dephasing coherent parameters. Thus, when this scheme is experimentally realized, it will be immune to collective decoherence (nonunitary process between the system and the environment) and robust against the decoherence effect (photon loss and dephasing in the interaction of XKNLs).
3. In our scheme, the designed nonlinearly optical gates employ qubus beams (two 50:50 BSs)^11,43,47^, and the strategy of PNR measurement^7,11,31,34,55,56,62,64,65^ for the controlled operation. If we use the displacement operator^7,55,56^ or homodyne measurement^36,37,54–56^ (instead of qubus beams or PNR measurement) for the same operation as nonlinearly optical gates (1st, 2nd, 3rd, and final), we should employ the conditional phase shift −θ (by XKNL: negative), as described in^62^. However, Kok^80^ showed that it is generally not possible to change the sign of the conditional phase shift (−θ). Thus, our nonlinearly optical gates (1st, 2nd, 3rd, and final) using only positive XKNLs (θ) with qubus beams and PNR measurement are more experimentally feasible than other gates^7,33–37,55,56^ that should to use the negative XKNL.
4. In particular, through our analysis in Sec. 3, we demonstrated that the nonlinearly optical gates in optical fibers can obtain high efficiency and reliable performance by increasing the amplitude of the coherent state (probe beam) under the decoherence effect (photon loss and dephasing). This also brings an experimental merit of the weak magnitude of an XKNL (θ) by due to increasing the amplitude of the coherent state in nonlinearly optical gates to obtain robustness against the decoherence effect (i.e., when *α* = 1 × 10^6^, the magnitude of XKNL is required to be θ = 2.5 × 10^−6^, because of the fixed condition *αθ* = 2.5 for ${{\rm{P}}}_{{\rm{err}}} < {10}^{-3}$). Therefore, because of the difficulty of implementing a large magnitude in XKNLs^64,81,82^, our nonlinearly optical gates using XKNL can be realized in practice.
5. The probe beam of path a can be recycled for other nonlinearly optical gates, because PNR measurements are operated in the probe beam of path b in all nonlinearly optical gates (1st, 2nd, 3rd, and final). Namely, the unmeasured probe beam (${|{{\rm{\Lambda }}}_{t}^{m}\alpha \rangle }_{P}^{a}\,{\rm{or}}\,{|{{\rm{\Lambda }}}_{t}^{m}\alpha \,\cos \,{\rm{\theta }}\rangle }_{{\rm{P}}}^{{\rm{a}}}$ where photon loss, ${{\rm{\Lambda }}}_{t}^{m}={e}^{-m\gamma t/2}$: photon loss rate according to nonlinearly optical gates) of path a can be utilized as the input probe beam (recycled) of other nonlinearly optical gates using qubus beams and XKNLs.

Consequently, we demonstrate that our scheme for generating single logical qubit information (into three-photon decoherence-free states) with the immunity (against collective decoherence between the system and the environment) can be experimentally implemented and be robust (against the decoherence effect in optical fibers) through our analysis of nonlinearly optical gates using XKNLs, qubus beams, and PNR measurements.

## Electronic supplementary material

Appendix
