# Supplementary material for: Preparation of quantum information encoded on three-photon decoherence-free states via cross-Kerr nonlinearities
Source: Sci Rep. 2018 Sep 14;8:13843. doi: 10.1038/s41598-018-32137-3 (PMC6138704; doi:10.1038/s41598-018-32137-3)
Supplement: Supplementary file 1 — Appendix [file 41598_2018_32137_MOESM1_ESM.docx]

**Title: Preparation of quantum information encoded on three-photon decoherence-free states via cross-Kerr nonlinearities**

Authors: Jino Heo, Min-Sung Kang, Chang-Ho Hong, Jong-Phil Hong, Seong-Gon Choi^*^

**APPENDIX**

We show the process model of the interaction of XKNL, $\tilde{X}_{t}$, and decoherence, $\tilde{D}_{t}$ (photon loss and dephasing) [55, 56, 62], from Eqs. 3 and 14, to analyze the decoherence effect in our gates. For a simple example, in Fig. A1, let us assume that the initial state, $|R\rangle\otimes|\alpha\rangle$, and the simple gate of XKNL (one times of controlled phase shift), in Fig. A1, are prepared.


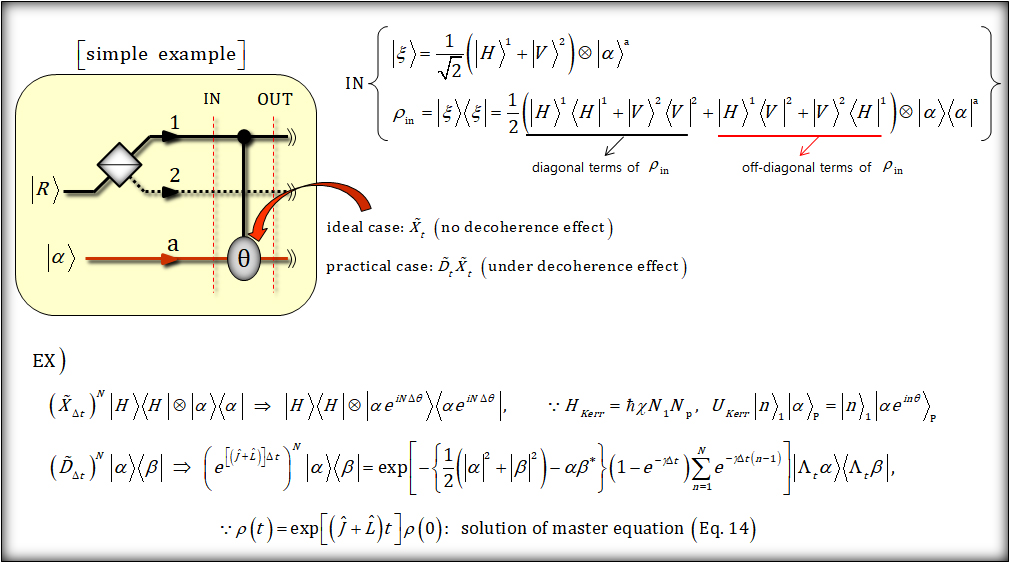


**Fig. A1.** Plot represents the simple example to show the process model of the interaction of XKNL, $\tilde{X}_{t}$, and decoherence, $\tilde{D}_{t}$ (photon loss and dephasing) [55, 56, 62]. And the equations in (EX) mean the interaction of XKNL from Kerr medium (Eq. 3) and the evolving process, due to the decoherence effect, by master equation (Eq. 14).

After PBS [red dotted line (IN) in Fig. A1], the initial state will be $|\xi\rangle$ and can be also formed as $\rho_{\text{in}}$, as shown in Fig. A1. For a good approximation of analysis [55, 56, 62], we can take a small time $\Delta t\text{ }\left(=t/N\right)$ for $\theta=\chi t=\chi N\Delta t=N\Delta\theta$.

**The Ideal case (no decoherence effect):** because of the only interaction, , of XKNL (controlled phase shift), we can obtain the output state, $\rho_{\text{out}}^{\text{idea}}$, [red dotted line (OUT) in Fig. A1], as follows:

$\begin{matrix} \rho_{\text{in}}\text{ }\mathop{\Rightarrow}\limits^{\text{XKNL}} \text{ }\tilde{X}_{t}\rho_{\text{in}}\equiv\left(\tilde{X}_{\Delta t}\right)^{N}\rho_{\text{in}}=\rho_{\text{out}}^{\text{ideal}}= \\ \text{ }=\frac{1}{2}\left(|H\rangle\langle H|^{1}\otimes|\alpha e^{i\theta}\rangle\langle\alpha e^{i\theta}|^{\text{a}}+|V\rangle\langle V|^{2}\otimes|\alpha\rangle\langle\alpha|^{\text{a}}+|H\rangle^{1}\langle V|^{2}\otimes|\alpha e^{i\theta}\rangle\langle\alpha|^{\text{a}}+|V\rangle^{2}\langle H|^{1}\otimes|\alpha\rangle\langle\alpha e^{i\theta}|^{\text{a}}\right), \end{matrix}$ (A1)

where $\theta=\chi t=\chi N\Delta t=N\Delta\theta$ for $\Delta t\text{ }\left(=t/N\right)$ and $\alpha\in\Re$. The result, $\rho_{\text{out}}^{\text{idea}}$, of Eq. A1 depends on the interactions of XKNL as

$\begin{matrix} \left(\tilde{X}_{\Delta t}\right)|H\rangle\langle H|^{1}\otimes|\alpha\rangle\langle\alpha|^{\text{a}}\text{ }\Rightarrow\text{ }|H\rangle\langle H|^{1}\otimes|\alpha e^{i\Delta\theta}\rangle\langle\alpha e^{i\Delta\theta}|^{\text{a}},\text{ }\left(\tilde{X}_{\Delta t}\right)|V\rangle\langle V|^{2}\otimes|\alpha\rangle\langle\alpha|^{\text{a}}\text{ }\Rightarrow\text{ }|V\rangle\langle V|^{2}\otimes|\alpha\rangle\langle\alpha|^{\text{a}}, \\ \left(\tilde{X}_{\Delta t}\right)|H\rangle^{1}\langle V|^{2}\otimes|\alpha\rangle\langle\alpha|^{\text{a}}\text{ }\Rightarrow\text{ }|H\rangle^{1}\langle V|^{2}\otimes|\alpha e^{i\Delta\theta}\rangle\langle\alpha|^{\text{a}},\text{ }\left(\tilde{X}_{\Delta t}\right)|V\rangle^{2}\langle H|^{1}\otimes|\alpha\rangle\langle\alpha|^{\text{a}}\text{ }\Rightarrow\text{ }|V\rangle^{2}\langle H|^{1}\otimes|\alpha\rangle\langle\alpha e^{i\Delta\theta}|^{\text{a}}, \end{matrix}$ (A2)

where the each interaction $\tilde{X}_{\Delta t}$ is sequentially operated $N$ times. Also the output state, $\rho_{\text{out}}^{\text{idea}}$ can be written to

$\rho_{out}^{ideal}=\frac{1}{2}\left(\begin{matrix} 1 & 1 \\ 1 & 1 \end{matrix}\right),$ (A3)

where we define the bases of density matrix as $\left\{\text{ }|H\rangle^{1}\otimes|\alpha e^{i\theta}\rangle^{a}\text{ },\text{ }|V\rangle^{2}\otimes|\alpha\rangle^{a}\text{ }\right\}$. And it is definitely possible to transform this density matrix, $\rho_{\text{out}}^{\text{idea}}$, to the pure state, $|\zeta\rangle_{\text{out}}$, due to no decoherence effect (ideal case).

$\rho_{\text{out}}^{\text{ideal}}=|\zeta\rangle\langle\zeta|_{\text{out}}\text{ }\Rightarrow\text{ }|\zeta\rangle_{\text{out}}=\frac{1}{\sqrt{2}}\left(|H\rangle^{1}\otimes|\alpha e^{i\theta}\rangle^{\text{a}}+|V\rangle^{2}\otimes|\alpha\rangle^{\text{a}}\right),$ (A4)

where the output state is still maintained the pure state, $|\zeta\rangle_{\text{out}}$, after the interaction, according to no dephasing and photon loss. Namely, this means that the absolute values of coherent parameters (off-diagonal terms) in density matrix ($\rho_{\text{out}}^{\text{idea}}$) remain 1, and we can find out no evolution to mixed state in the ideal case (no decoherence effect).

However, we should consider the decoherence effect (dephasing and photon loss) to realize quantum information processing scheme, and to acquire the feasibility of quantum controlled operation, in practice. From solving the master equation, which can be described the open quantum system, we show the processing model [55, 56, 62] of the interaction of XKNL, $\tilde{X}_{t}$, and decoherence, $\tilde{D}_{t}$ (photon loss and dephasing), to analyze the dephasing of coherent parameters and photon loss in coherent state (probe beam), and also to obtain the requirement for the robustness against the decoherence effect in our nonlinearly optical gates (1st, 2nd, 3rd, and final).

**The practical case (under decoherence effect):** when to operate the interaction, , of XKNL between a photon and coherent state in Kerr medium, as described in Fig. A1, the decoherence, , simultaneously occurs in the process of simple example. Therefore, after the interaction of the same input state, $\rho_{\text{in}}=|\xi\rangle\langle\xi|$, we can calculate the output state, $\rho_{\text{out}}^{\text{pract}}$, [red dotted line (OUT) in Fig. A1] by the solution of Eq. 14, and Eq. 3 (XKNL), as follows:

$\begin{matrix} \rho_{\text{in}}\text{ }\mathop{\Rightarrow}\limits^{\text{XKNL + decoherence}} \\ \text{ }\Rightarrow\text{ }\tilde{D}_{t}\tilde{X}_{t}\rho_{\text{in}}\equiv\left(\tilde{D}_{\Delta t}\tilde{X}_{\Delta t}\right)^{N}\rho_{\text{in}}=\rho_{\text{out}}^{\text{pract}} \\ \text{ }=\frac{1}{2}\left[\left[\text{ }|H\rangle\langle H|^{1}\otimes|\Lambda_{t}\alpha e^{i\theta}\rangle\langle\Lambda_{t}\alpha e^{i\theta}|^{\text{a}}+|V\rangle\langle V|^{2}\otimes|\Lambda_{t}\alpha\rangle\langle\Lambda_{t}\alpha|^{\text{a}}\right.+\right. \\ \text{ }+\exp\left[-\text{ }\alpha^{2}\left(1-e^{-\gamma\Delta t}\right)\sum_{n=1}^{N} e^{-\gamma\Delta t\left(n-1\right)}\left(1-e^{+in\Delta\theta}\right)\right]|H\rangle^{1}\langle V|^{2}\otimes|\Lambda_{t}\alpha e^{i\theta}\rangle\langle\Lambda_{t}\alpha|^{\text{a}}+ \\ \text{ }\left.\left.+\exp\left[-\text{ }\alpha^{2}\left(1-e^{-\gamma\Delta t}\right)\sum_{n=1}^{N} e^{-\gamma\Delta t\left(n-1\right)}\left(1-e^{-in\Delta\theta}\right)\right]|V\rangle^{1}\langle V|^{2}\otimes|\Lambda_{t}\alpha\rangle\langle\Lambda_{t}\alpha e^{i\theta}|^{\text{a}}\text{ }\right]\right], \end{matrix}$ (A5)

where $\Lambda_{t}=e^{-\gamma t/2}$ is photon loss rate in the coherent state, and $\theta=\chi t=\chi N\Delta t=N\Delta\theta$ for $\Delta t\text{ }\left(=t/N\right)$. The result, $\rho_{\text{out}}^{\text{pract}}$, of Eq. A5 depends on the interactions of XKNL (as Eq. A2) and of decoherence as

$\begin{matrix} \left(\tilde{D}_{\Delta t}\right)|\alpha e^{i\phi}\rangle\langle\beta e^{i\varphi}|\text{ }\Rightarrow\text{ }\left(e^{\left[\left(\hat{J}+\hat{L}\right)\right]\Delta t}\right)|\alpha\rangle\langle\beta|\text{ }\Rightarrow\text{ } \\ \text{ }\Rightarrow\text{ }\exp\left[-\left\{\frac{1}{2}\left(\left|\alpha e^{i\phi}\right|^{2}+\left|\beta e^{i\varphi}\right|^{2}\right)-\alpha\beta^{*}e^{i\left(\phi-\varphi\right)}\right\}\left(1-e^{-\gamma\Delta t}\right)\right]|e^{-\gamma\Delta t/2}\alpha e^{i\phi}\rangle\langle e^{-\gamma\Delta t/2}\beta e^{i\varphi}|, \end{matrix}$ (A6)

where $\tilde{D}_{t}$ is the solution of master equation (Eq. 14). For a good approximation, we have chosen the interaction time $\Delta t$ that divides into the whole time, $t$, $N$ times. Thus, the whole interaction $\tilde{X}_{t}$ and $\tilde{D}_{t}$ (during time $t$) is composed by each interaction $\tilde{X}_{\Delta t}$ and $\tilde{D}_{\Delta t}$ (during time $\Delta t$) $N$ times in series. Therefore, we show the procedure of this interaction $\tilde{X}_{t}$ and $\tilde{D}_{t}$ to derive the output state, $\rho_{\text{out}}^{\text{pract}}$ in Eq. A5, as follows:

(A7)

where this interaction ($\tilde{X}_{\Delta t}$ and $\tilde{D}_{\Delta t}$) is in the first step for time $\Delta t$. The state, $\rho_{\text{in}}^{\text{PS1}}$, is only interacted with XKNL (phase shift: $\Delta\theta$ during $\Delta t$), $\tilde{X}_{\Delta t}$ in Eq. A2. After the decoherence ($\tilde{D}_{\Delta t}$ in Eq. A6) applies to the state $\rho_{\text{in}}^{\text{PS1}}$, we can obtain the output state $\rho_{\text{in}}^{\text{pract\_1}}$ of the first step. Then, the second step (sequentially operated) is given by

(A8)

Subsequently, the processes of the remain steps (3rd ~ $N$th step) are calculated by the same formula $\left(\tilde{D}_{\Delta t}\tilde{X}_{\Delta t}\right)$ of Eq. A7 or A8. Consequently, after to operate the $N$th step (after the interaction $\tilde{X}_{\Delta t}$ and $\tilde{D}_{\Delta t}$ $N$ times), which means $\tilde{D}_{t}\tilde{X}_{t}=\left(\tilde{D}_{\Delta t}\tilde{X}_{\Delta t}\right)^{N}$, the output state will be finally given as $\rho_{\text{out}}^{\text{pract}}\text{ }\left(=\rho_{\text{in}}^{\text{pract\_}N}\right)$ in Eq. A5 where $e^{-\gamma t/2}=e^{-\gamma N\Delta t/2}$ and $\alpha e^{iN\Delta\theta}=\alpha e^{i\theta}$, according to $\theta=\chi t=\chi N\Delta t=N\Delta\theta$ and $\Delta t\text{ }\left(=t/N\right)$.

So far we have shown the procedure (from 1st to $N$th step) of calculation for the output state ($\rho_{\text{out}}^{\text{pract}}$ in Eq. A5) applied the interactions of $\tilde{X}_{t}$ and $\tilde{D}_{t}$. In Eq. A5 ($\rho_{\text{out}}^{\text{pract}}$), we define the coherent parameter (coefficient of off-diagonal terms) as $\text{T}\equiv\exp\left[-\text{ }\alpha^{2}\left(1-e^{-\gamma\Delta t}\right)\sum_{n=1}^{N} e^{-\gamma\Delta t\left(n-1\right)}\left(1-e^{+in\Delta\theta}\right)\right]$. Through this value, $\text{T}$, of coherent parameter and photon loss rate, $\Lambda_{t}$, we can analyze and quantify the amount of dephasing (evolving to mixed state: fidelity) and photon loss (the error probability according to the measurement strategies: efficiency). The density matrix, $\rho_{\text{out}}^{\text{pract}}$, of the output state is given by

$\rho_{out}^{pract}=\frac{1}{2}\left(\begin{matrix} 1 & T \\ T^{*} & 1 \end{matrix}\right),$ (A9)

where we also define the bases of density matrix, $\rho_{\text{out}}^{\text{pract}}$, as $\left\{\text{ }|H\rangle^{1}\otimes|\Lambda_{t}\alpha e^{i\theta}\rangle^{a}\text{ },\text{ }|V\rangle^{2}\otimes|\Lambda_{t}\alpha\rangle^{a}\text{ }\right\}$. If the dephasing of coherent parameter doesn’t exist in the interaction between a photon and coherent state, the density matrix, $\rho_{\text{out}}^{\text{pract}}$, can be written as

$\rho_{\text{out}}^{\text{pract}}=|\zeta'\rangle\langle\zeta'|_{\text{out}}\text{ }\mathop{\Rightarrow}\limits^{\text{no dephasing}} \text{ }|\zeta'\rangle_{\text{out}}=\frac{1}{\sqrt{2}}\left(|H\rangle^{1}\otimes|\Lambda_{t}\alpha e^{i\theta}\rangle^{\text{a}}+|V\rangle^{2}\otimes|\Lambda_{t}\alpha\rangle^{\text{a}}\right),$ (A10)

where the only photon loss affects to the output state, $|\zeta'\rangle_{\text{out}}$ (pure state). However, as mentioned above, in the practical case, the decoherence effect (dephasing and photon loss) is unavoidable when to operate the interaction a photon and coherent state in optical fiber. Thus, under the decoherence effect (invariably evolving to mixed state), this expression (Eq. A10) of the pure state is unavailable (different from the ideal case) to quantify the decoherence effect, due to the coherent parameters, involved with dephasing, of off-diagonal terms. Consequently, we should handle the density matrix, as Eq. A9, or mixed state (the linear combination of ensembles), as Eq. A5, of the output state in the optical gate to analyze the amount of dephasing (evolving to mixed state: fidelity) and photon loss (the error probability according to the measurement strategies: efficiency).

In section 3, for the analysis of nonlinearly optical gates (1st, 2nd, 3rd, and final) using XKNLs, qubus beams, and PNR measurement (under the decoherence effect), we will utilize the process model of the practical case (Eqs. A5 and A9) via the interaction of XKNL, $\tilde{X}_{t}$, and the solution, $\tilde{D}_{t}$, of master equation (Eq. 14).
